# “Salivary gland cellular architecture in the Asian malaria vector mosquito *Anopheles stephensi*”

**DOI:** 10.1186/s13071-015-1229-z

**Published:** 2015-12-02

**Authors:** Michael B. Wells, Deborah J. Andrew

**Affiliations:** Department of Cell Biology, Johns Hopkins University School of Medicine, 725 N. Wolfe St., G-10 Hunterian, Baltimore, MD 21205 USA

**Keywords:** *Anopheles*, Salivary gland, Malaria, *Drosophila*, Cell architecture, Secretion

## Abstract

**Background:**

*Anopheles* mosquitoes are vectors for malaria, a disease with continued grave outcomes for human health. Transmission of malaria from mosquitoes to humans occurs by parasite passage through the salivary glands (SGs). Previous studies of mosquito SG architecture have been limited in scope and detail.

**Methods:**

We developed a simple, optimized protocol for fluorescence staining using dyes and/or antibodies to interrogate cellular architecture in *Anopheles stephensi* adult SGs. We used common biological dyes, antibodies to well-conserved structural and organellar markers, and antibodies against *Anopheles* salivary proteins to visualize many individual SGs at high resolution by confocal microscopy.

**Results:**

These analyses confirmed morphological features previously described using electron microscopy and uncovered a high degree of individual variation in SG structure. Our studies provide evidence for two alternative models for the origin of the salivary duct, the structure facilitating parasite transport out of SGs. We compare SG cellular architecture in *An. stephensi* and *Drosophila melanogaster*, a fellow Dipteran whose adult SGs are nearly completely unstudied, and find many conserved features despite divergence in overall form and function. *Anopheles* salivary proteins previously observed at the basement membrane were localized either in SG cells, secretory cavities, or the SG lumen. Our studies also revealed a population of cells with characteristics consistent with regenerative cells, similar to muscle satellite cells or midgut regenerative cells.

**Conclusions:**

This work serves as a foundation for linking *Anopheles stephensi* SG cellular architecture to function and as a basis for generating and evaluating tools aimed at preventing malaria transmission at the level of mosquito SGs.

**Electronic supplementary material:**

The online version of this article (doi:10.1186/s13071-015-1229-z) contains supplementary material, which is available to authorized users.

## Background

Mosquito transmitted disease represents a major threat to human health. Hundreds of millions of infections occur each year, leading to nearly two million deaths. The majority of these deaths are caused by malaria transmitted by mosquitoes of the genus *Anopheles*. Thirty-nine species of *Anopheles* are known to contribute to malaria infection worldwide [1], and two of the major vector species are *Anopheles gambiae* (prevalent in Africa) and *Anopheles stephensi* (prevalent in India). These are also two of the most well-studied mosquito species.

The life cycle of malaria parasites, *Plasmodium species*, has been characterized [2–5]. The parasite is acquired by mosquitoes that blood feed on infected humans [3]. Parasite gametes fuse inside the mosquito midgut to form zygotes that mature into motile ookinetes, which traverse the peritrophic matrix and midgut epithelium to form an oocyst in the gut wall lining [6]. Within the oocyst, the parasites multiply and mature into sporozoites, which travel via hemolymph flow to the salivary glands (SGs) after oocyst rupture. *Plasmodium* sporozoites acquire the ability to infect mammalian liver cells either in the hemolymph [7] or in the SGs [8]. Twenty percent of parasites that escape the midgut enter the SGs [5, 9, 10], while the rest are cleared from the mosquito. SG invasion is thought to involve receptor/ligand interactions; several parasite coat proteins (CSP, MAEBL, TRAP, UOS3, CRMP1/2), as well as SG surface sugar molecules (e.g. heparin sulfate) and proteins (SGS1, Saglin, TRAP) have been implicated in this process [4]. Once sporozoites contact the SGs, the parasite is thought to traverse the basement membrane via gliding motility and invade the SG epithelial cell by a process similar to cell engulfment, using the plasma membrane to form a second outer membrane (parasitophorous vacuole), which is subsequently lost. The parasite exits the epithelial cell into the secretory cavity, where hundreds to thousands of sporozoites collect. Only a small number of parasites can enter the salivary duct to be injected into their next host upon subsequent blood feeding. Parasites are injected along with mosquito saliva and a complement of factors that prevent clotting and host immune response [2, 3]. Despite over 100 years of discontinuous work focused on disease transmission to humans, mosquito biology at the cellular and molecular levels remains understudied.

Adult *An. stephensi* SG morphology has been described using electron microscopy (EM) [11, 12], where a number of observations regarding cell shape, organelle localization, and secretion characteristics were made. Other accounts of *Anopheles* adult SG structure by light and fluorescence microscopy have illuminated additional details regarding gross morphology, but these studies are quite limited in scope [13–16]. In contrast, a number of labs have characterized the proteins produced in *Anopheles* SGs, either *en masse* through mass spectrometry [17–20], or individually through biochemistry and molecular genetics methods [21–23]. Results overlap as far as the salivary proteome at large is concerned, but studies of proteins at the cellular level, particularly of protein localization by immunofluorescence, have produced inconsistent results and are typically limited to examination of a single protein [24–30]. One group has also recently generated *Anopheles stephensi* RNA-seq profiles at many developmental stages, with representative time points from early embryogenesis through early adulthood in either sex [31].

The limited characterization of adult SGs is not a problem unique to *Anopheles* and other insect vectors of disease. Indeed, very little is known regarding adult SG architecture in *Drosophila melanogaster*, a major model organism in laboratory research. Aside from a study of microfilament and microtubule organization [32], almost nothing has been done to characterize *Drosophila* adult SGs. Several accounts exist of conservation of function between *Drosophila* and *Anopheles* at the levels of epigenetic regulation, RNA, DNA, and protein. Marhold et al. showed that a DNA methyltransferase and its modification are conserved across Dipteran species [33]. Sieglaf et al. found 18 families of conserved cis regulatory elements among four Dipteran species [34]. Ahanger et al. found that *Anopheles* Hox gene boundary elements function as potent insulators in *D. melanogaster* [35]. Yoder and Carroll determined that the function of the posterior Hox gene AbdB is conserved across Diptera, despite a gene duplication and specialization that occurred in *Drosophila* [36]. Zdobnov and colleagues compared the proteomes of *An. gambiae* and *D. melanogaster*, showing a high degree of conservation [37]. In sum, this work underscores the high degree of conservation observed across Diptera, even among widely divergent species, as well as an emerging understanding of the *An. stephensi* genome and transcriptome.

Our aim is to better understand cellular architecture and secretion in *An. stephensi* SGs, a tissue critical for parasite transmission. We chose to focus on adult mosquitoes 7 days post emergence, when SG maturation is thought to be complete and secretion active [38]. We find key structural proteins, organelle markers, and several transcription factors to be highly conserved between mosquitoes and fruit flies (both of the order Diptera). The extent of conservation is somewhat remarkable, given 270 million years divergence between *An. stephensi* and *D. melanogaster* [39]. Divergence time is large even within the genus; about 60 million years separate *An. stephensi* and *An. gambiae* [39]. Using a panel of structural and organelle marker antibodies produced against *Drosophila* proteins, and antibodies against several *Anopheles* SG protein products, we applied an improved immunofluorescence protocol to day 7 adult *An. stephensi* SGs. Using immunofluorescence allows us to process many more samples than electron microscopy. Our results agree with prior studies of overall *An. stephensi* SG architecture; we note several key differences, however, as well as additional novel findings. We find the majority of *An. stephensi* male SGs to be branched, not largely mono-lobed, as was previously described in *Anopheles* [13]. We find several SG protein products to localize primarily within the SGs, instead of largely on the gland membrane surface [24, 25]. We observe cases of sexual ambiguity in salivary duct morphology in extranumerary female lobes. Finally, we identify a novel population of cells, which contain a small DAPI body and many cellular components. These cells have a high nuclear to cytoplasmic volume ratio and may represent mosquito SG secretory cell precursors. Altogether, this work highlights the major conserved features and variation of SG cellular architecture within *Anopheles stephensi*, confirms the utility of our revised immunofluorescence protocol, and lays the foundation for further studies of *Anopheles stephensi* SG interactions with malaria parasites at the cellular level.

## Methods

### Mosquito husbandry

Adult *Anopheles stephensi* (Dutch strain) were obtained from the Johns Hopkins Bloomberg School of Public Health Insectary, where they are maintained at 28 °C on 10 % sucrose *ad libitum* using standard procedures [40].

### Dissection and storage

Adult mosquitoes aged 7 days post eclosion were collected from cages as needed using a flashlight vacuum. The adults were knocked down at −23 °C for approximately 5 min, then kept in a petri dish on ice while dissections occurred. Individual adults were transferred from the petri dish to a six well dissection plate filled with 1X PBS. The heads and attached SGs were removed from the body by gripping the ventral thorax (non-dominant hand) and the base of the proboscis (dominant hand) using fine tip tweezers and gently pulling in opposite directions with minimal constant force. Heads and SGs were then transferred to microcentrifuge tubes on ice containing 1X PBS, where they were gently placed on top of the buffer. Any submerged tissue was discarded, since excess internal liquid was found to harm the tissue upon freezing. Aliquots of ten heads were either processed at this time or transferred to −80 °C for long term storage. SGs frozen in this manner remained suitable for analysis for up to 1 month following dissection.

### *Anopheles stephensi* fixation, immunofluorescence, and mounting

Aliquots of heads/SGs were either processed fresh or thawed from −80 °C to room temperature. Immediately after, the PBS was removed. 400 μL cold absolute acetone was added, and the tube partially inverted twice. The tissue was fixed in acetone at room temperature for 90 s. The acetone was then removed and replaced by 200 μL 1X PBS and incubated at room temperature for 30 min. The PBS was then removed and replaced with diluted primary antibody in PBS and incubated overnight at 4 °C without shaking. The primary antibody solution was removed and the glands washed briefly in 1X PBS the following day. The PBS was removed and replaced with 1:100 secondary antibody (Life Technologies) in PBS and incubated in the dark at room temperature for 2 h. Then, the secondary antibody solution was removed and the glands were incubated for 30 min in the dark at room temperature with a 1:60 dilution of DAPI in 1X PBS. This solution was then removed, the glands were briefly washed in 1X PBS two to four times, and a final 200 μL 1X PBS was added. Samples were stored in the dark at 4 °C until mounting (up to 1 week later).

We noticed little to no signal from mouse secondary antibody alone staining (Additional file 1: Figure S4); however, low level salivary duct and perinuclear accumulations were observed in rabbit secondary antibody alone staining experiments, regardless of the fluorophore used (Additional file 1: Figure S4; data not shown). Addition of rabbit primary antibodies greatly reduced this background signal, to the point where it was not observed with most rabbit primary antibodies.

Formaldehyde fixation alone was found to be ineffective for tissue penetration by the antibody. Formaldehyde fixation for 2 h in the presence of 1:20 glacial acetic acid (added 15 s after the formaldehyde and gently mixed) worked better. Finally, a 60 s incubation in 45 % acetic acid also provided adequate SG tissue fixation. The last two methods were not often used, however, because those methods were not as dependable as acetone fixation.

Stained glands were transferred from the microcentrifuge tube to a VWR superfrost plus microscope slide using a 200 μL pipette tip. 100 μL of 100 % glycerol was added to the sample. Glands were then positioned on the slide under glycerol using fine or ultra-fine-tipped forceps and/or sectioned away from the head using tungsten needles (0.125 mm, Fine Science Tools). A 20x40 coverslip was then placed on top of the sample in glycerol. Slides were kept covered at 4 °C and samples could be imaged for up to 2 months.

### Drosophila melanogaster fixation, immunofluorescence, and mounting

Third instar *Drosophila melanogaster* (Oregon R strain) larval SGs were dissected and incubated in cold absolute acetone for 90 s. Samples were stained with DAPI overnight, rinsed, mounted under glycerol, and visualized.

Adult *Drosophila melanogaster* (Canton S strain) were anesthetized with carbon dioxide. Individual adults were transferred to the well edges of a six well glass dissection plate containing 1X PBS. With the fly positioned dorsal up, the head and SGs were removed by placing tweezers over the abdomen and just behind the neck and gently, but swiftly, pulling off the head in one motion. Frequently, the gut and crop had to be manually severed and removed. The head and SGs were placed on top of 1X PBS in a microcentrifuge tube until dissections were complete. Females and males were dissected in sex-specific groups, and their tissues were kept separate, to allow for easy identification of sex later. Staining was performed as for *An. stephensi*, except that all washes were completed with PBSTB (0.1 % Triton X-100, 0.2 % BSA) instead of PBS.

### Dyes and antibodies

Lamin C and α-tubulin antibodies were purchased from the Developmental Studies Hybridoma Bank (DSHB). LC28.26 (Lamin C) was deposited to the DSHB by Fisher, P.A. (DSHB Hybridoma Product LC28.26). 6G7 (α-tubulin) was deposited to the DSHB by Halfter, Willi M. (DSHB Hybridoma Product 6G7). See Table 1 and in-text references for information about the dyes and other antibodies used in this study.

**Table 1:** Antibody/dye information and usage

Dye/Target protein	Target species	Host	Dilution	Epitope	Source
DAPI	n/a	n/a	1:60	n/a	Life Technologies
Rh-WGA	n/a	n/a	1:40	n/a	Vector Labs
Nile Red	n/a	n/a	1:60	n/a	Sigma
phalloidin-488	n/a	n/a	1:10	n/a	Life Technologies
AAPP	*An. stephensi*	rabbit	1:100	AA 26-293	Matsuoka lab
SG6	*An. gambiae*	mouse	1:100	unknown	Lombardo lab
mtTFA	human	rabbit	1:20	AA 44-246	Santa Cruz (H-203)
KDEL receptor	bovine	mouse	1:20	full length	Abcam ab69659
GM130	*Drosophila*	rabbit	1:20	C-terminus	Abcam ab30637
α-tubulin	chicken	mouse	1:10	unpublished	DSHB (clone AA4.3)
Lamin C	*Drosophila*	mouse	1:5	full length	DSHB (clone LC28.26)

### Confocal microscopy

Slides were imaged with either a Zeiss LSM700, Zeiss LSM780, or Zeiss Meta 510 confocal microscope at 20X, 40X, or 100X (under oil) magnification as 3-dimensional z-stacks with a step-size of one micron. Single slice images were shown in the figures unless otherwise noted. Zeiss Zen 2012 was used for the creation of 3D projections, scale bar addition, and image contrast optimization. Scale bars were added in Zen, then used as a template for final scale bar addition in Microsoft PowerPoint.

### Homology search

Drosophila protein sequences were obtained from Flybase [41] and inputted into NCBI BLASTp [42] to detect closely related protein sequences in *An. stephensi* (derived from AsteS2 gene set). BLAST statistics comparing *An. stephensi* to *D. melanogaster* or to *An. gambiae* were generated and are presented in Tables 2 and 3. All homologous gene calls were confirmed by cross-referencing to listed *Anopheles* homologs for *Drosophila* genes found on Flybase. Determining homologs by BLASTp search gave us a quantitative measure of homology/identity within each *Anopheles* homolog, providing us with evidence either for or against the expectation that staining with a particular antibody would work.

**Table 2:** Conservation of selected organelle or structural markers in *Anopheles stephensi*

Gene		% Coverage of *Drosophila*	% Max identity	*E*-value
*D. melanogaster*	*An. stephensi*			
*α-Tubulin*	ASTE006004	100	99	0
KDEL receptor	ASTE003703	100	83	2.00E-127
*Lamin*	ASTE007371	100	49	0
*mtTFA*	ASTE007783	86	31	1.00E-39
*GM130*	ASTE011240	85	34	3.00E-88

**Table 3:** Conservation of selected proteins between two *Anopheles* species

Gene			% Coverage of *An. gambiae*	% Max identity	*E*-value
	*An. stephensi*	*An. gambiae*			
*α-Tubulin*	ASTE006004	AGAP001219	100	100	0
KDEL receptor	ASTE003703	AGAP010224	100	100	5.00E-157
“		AGAP012756	100	100	5.00E-157
Lamin C	ASTE007371	AGAP011938	100	84	0
AAPP	ASTE004273	AGAP009974	100	55	7.00E-85
SG6	ASTE000264	AGAP000150	99	75	7.00E-64
*GM130*	ASTE011240	AGAP011337	98	77	0
*mtTFA*	ASTE007783	AGAP008499	98	82	4.00E-158

### Multiple sequence alignments and conservation tree diagrams

Additional file 1: Figures S5–S10 (A) show multiple sequence alignments of homologs from *An. gambiae* (AGAP…), *An. stephensi* (ASTE…), *D. melanogaster* (FBgene…), *An. darlingi* (ADAC…), *An. albimanus* (AALB…), *An. quadriannulatus* (AQUA…), *An. arabiensis* (AARA…), *An. funestus* (AFUN…), *An. dirus* (ADIR…), and *Aedes aegypti* (AAEL…) created using the CLUSTALW and BOXSHADE tools at the SDSC Biology Workbench [43]. Protein sequences were obtained from Vectorbase [44], Uniprot [45], and Flybase [41]. Green shading denotes perfect conservation, teal indicates sites with conservative substitutions (according to the following groupings—FYW IVLM RK DE GA TS NQ), and yellow indicates residues that are the same at that position in at least 50 % of the sequences analyzed; see [46] and the SDSC Biology Workbench (http://workbench.sdsc.edu/) for more information about classifications.

Parts (B) of Additional file 1: Figures S5 and S10 show protein conservation tree diagrams aligned by gene structure, obtained from Vectorbase [44]. A key is provided to explain node color identity, contrast homologs and paralogs, and explain conservation shading within the diagram.

### Morphometry

Images of mosquito SGs that included a suitable membrane marker (such as Rh-WGA or Nile Red staining) were interrogated for lobe length, lobe width, duct width, cell length, and cell width using the 3D_Distance_Tool macro in ImageJ [47]. Ten samples were measured per lobe dimension and fifty samples per cell dimension. Cross-section images for circumferential nuclei counts were generated and analyzed in Zeiss Zen 2012.

Drosophila larval SG nuclear diameters were collected using the 3D_Distance_Tool in ImageJ [47] and graphed as averages with standard deviations in Microsoft Excel 2013. Statistical analysis was conducted using Minitab 17.

## Results

### *Anopheles stephensi* adult salivary gland architecture

**Fig. 1:**
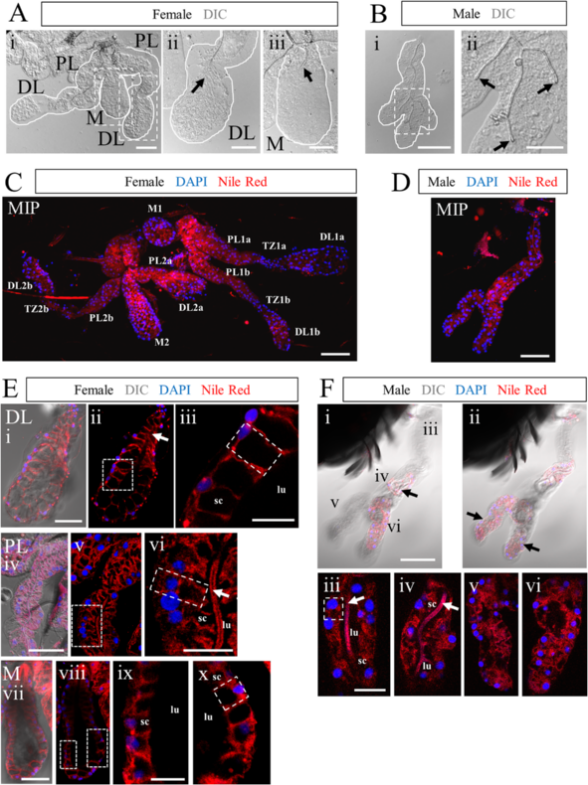
*Anopheles stephensi* salivary gland organization is elaborate and sexually dimorphic. Morphological overview of Anopheles stephensi adult SGs. Low magnification Normarski images of female (**a**) and male (**b**) SGs. The female gland comprises two lateral lobes, containing proximal lateral (PL), transition zone, and distal lateral (DL) regions, and a single medial lobe (M). Two glands are present in each mosquito, connected by a common duct. The male gland occurs as either a tube with several branches (more frequent, shown), or an unbranched linear tube (very rare morphology). Note that female duct termini are open, whereas male duct termini are fused (black arrows). **c**-**f** Confocal images of SGs stained with Nile red (membranes) and DAPI (nuclei). **c**-**d** Confocal maximum intensity projection (MIP) of female (**c**, all lobes/regions of two connected glands are labeled) or male (**d**) SGs. **e**-**f** Confocal slice images detailing the female SG lobes (**e**) and male gland (**f**) accompanied by higher magnification zoom images. Nile red staining highlights the cup-shaped cell structure surrounding a secretory cavity and cellular arrangement within each lobe. Narrow cell body domains, lateral to the secretory cavity, of two adjacent cells abut most of the time. Male SG ducts often terminate well before the distal-most regions (Fii). This correlates with distal region cellular disorganization, including the lack of a clear lumen (Fv-vi). More proximal regions are more often clearly arranged (similar to female lobes), but with smaller secretory cavities and less basally compressed cell bodies (Fiii). Numerous duct bifurcations are common in males (Fiii). The duct (Eii, Evi, Fiii, Fiv; white arrows) is weakly marked by Nile Red. Narrow solid white lines in low magnification fluorescent images indicate the limits of the gland/lobe based on DIC [not shown after (**a**)] or low level Nile Red staining. Thicker dotted lines indicate regions magnified in additional panels to the right. A single cell in each female lobe and in the male SG is outlined by a dashed box (1E-F). Scale bar lengths are: 50 microns—Ai, Bi, C, D, Ei, Eiv, Evii, Fi; 20 microns—Aii, Aiii, Bii, Eiii, Evi, Eix, Fiii. DL—distal lateral lobe; PL-proximal lateral lobe; M-medial lobe; TZ-lateral lobe transition zone

We first sought to validate previous studies of gland morphology by employing light and fluorescence microscopy to the SGs of our strain of *Anopheles stephensi*. In agreement with previous accounts, females had two glands (Fig. 1c), each composed of one medial lobe and two lateral lobes divided into proximal and distal regions (Fig. 1Ai). Male *An. stephensi* SGs were most commonly a single lobe that bifurcated at one or more point(s) along the central axis into two or three “lobes” of similar shape (Fig. 1Bi). A salivary duct ran throughout the SGs; in females, duct termini were primarily open (Fig. 1Aii-iii, *arrows*), and the duct extended deeper into the distal lateral lobes than the medial lobe (Fig. 1Aii-iii). Male salivary ducts also extended nearly the entire length of each “lobe”. In contrast to female salivary duct termini, male termini were almost always fused (Fig. 1Bii, *arrows*).

Using the lipophilic dye Nile Red, we interrogated *An. stephensi* SG cell shape and arrangement (Fig. 1c-f). Maximum intensity projections (MIP) of Nile Red and DAPI (nuclei) (Fig. 1c-d) revealed the details of cell arrangement. Nuclei and cell bodies were typically basally positioned, and cell distance from the duct/lumen varied, as did the shape of individual lobes. The cup shape of SG cells was readily observed in higher magnification images (Fig. 1e and f). The ordered arrangement of cells was highest in the distal lateral and medial lobes, where the cell bodies were basally flattened (Fig. 1Eiii, ix-x). The proximal lateral lobe cells were less basally compressed and secretory cavities appeared smaller (Fig. 1Eiv-vi). In male glands, cell shape was much less often cup-shaped and cells were sometimes disordered (Fig. 1Fiii, v-vi compared to Fig. 1Fiv), and lumen shape was frequently irregular (Fig. 1Fv-vi). In some instances (Fig. 1Eii, vi, Fiii-iv; *arrows*), the duct stained prominently with Nile Red.

Nile Red and DAPI staining were used to determine the dimensions of each lobe, cell size, and how many cells lie in circumference around the lumen in each lobe (Additional file 1: Figure S1). Lobe length varied between 150 and 400 microns; the female DL and M lobes were somewhat shorter than the female PL lobe and male SG (Additional file 1: Figure S1A; pair-wise Mann-Whitney U tests, *p* < 0.001). Lobe width was between 30 and 75 microns; the female DL and M lobes were wider than the female PL lobe and male SG (Additional file 1: Figure S1A; pair-wise Mann-Whitney U tests, *p* < 0.001). Cell length (along the apico-basal axis) was 25–30 microns in females and was about 10 microns on average in males (Additional file 1: Figure S1B). Average cell width in any lobe was about 10 microns (Additional file 1: Figure S1B). By obtaining cross-sectional views, the number of cells in circumference in each lobe was determined. Female gland values ranged from 6 to 14, whereas the male gland values ranged from 2 to 12, at different positions along the proximal distal axis of each gland/lobe (Additional file 1: Figure S1C.) These data demonstrate the utility of our staining methodology using two common biological fluorescent dyes.

#### Salivary duct architecture

**Fig. 2:**
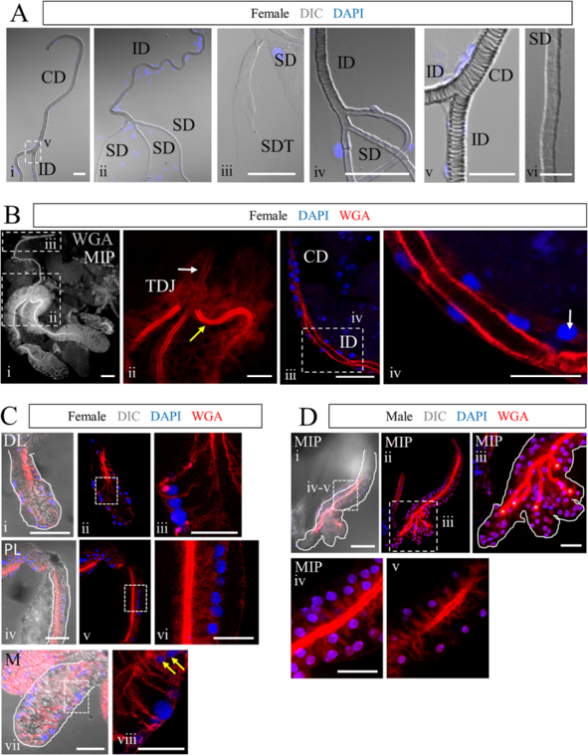
Duct appearance differs outside versus inside of the salivary glands. **a** DIC imaging of DAPI-stained *An. stephensi* female SGs highlighting the duct. Ladder-like taenidia are obvious in the portions of the duct proximal to the SG (Ai-ii, iv-v). Once inside the gland, duct morphology becomes smooth (Aii-iv, vi), continuing all the way through the termini (Aiii). **b**-**d** Confocal images of adult *An. stephensi* SG lobes stained with rhodamine-conjugated wheat germ agglutinin (WGA; chitin, O-GlcNAc groups) and DAPI (nuclei). (2Bi) Maximum intensity projection of WGA signal (greyscale) throughout an entire female SG as well as the individual duct (ID) and part of the common duct (CD) is shown. The triductal junction (TDJ) within the SG is highlighted (red) in the zoom image shown in Bii. Duct chitin levels are higher inside the SG (yellow arrow) than outside (white arrow). Biii (90^o^ counter-clockwise rotation of boxed region in Bi) shows one ID as it branches away from the CD. Biv is a zoom of the boxed region in Biii, highlighting SG duct organization. In the CD and ID, cells/nuclei are typically found in close association with the duct. A low percentage of individual CD and ID cells/nuclei are more tightly associated with the basement membrane (arrow in Biv). **c** Confocal images of female SGs stained with WGA and DAPI (nuclei). Rare nuclei are observed in close proximity to the duct (Dviii, yellow arrows). **d** Confocal images of male SGs stained with WGA and DAPI. WGA staining labels the chitinous salivary duct and O-GlcNAcylated proteins within SG cells. Five fused duct termini are marked by asterisks (Diii). Scale bar lengths are: 50 microns—Bi, Ci, Civ, Cvii, Di; 20 microns—Ai, Aiii, Aiv, Bii, Biii, Ciii, Cvi, Cviii, Diii, Div; 10 microns—Av, Biv; 5 microns: Avi. CD: common duct, ID: individual duct, SG: salivary gland, SD: salivary duct (in gland), SDT: salivary duct terminus, TDJ: triductal junction

The salivary duct is the passageway for parasite and virus transmission from the SGs to hosts. DIC and fluorescence imaging were employed to obtain better views of salivary duct structure (Fig. 2a). A common duct (CD) leads from the proboscis, past a pump muscle, to a branch point [48]. From there, two individual ducts (ID) split off and lead to each side of the mosquito, one to each SG. Once inside the gland, salivary ducts (SD) extended most of the distance of each lobe, terminating in the distal lateral and medial lobes in females and near the distal end of each lobe in males. DIC imaging of various regions of the duct stained with DAPI revealed a tight association of nuclei with the duct in the CD and ID, which was not observed in the SD (Fig. 2Aii, iv-v). Also, DIC images of the CD and ID revealed that the lumenal surfaces of the duct had a ringed ladder-like structure consistent with taenidia (Fig. 2Ai-ii, v), which have been previously described in the trachea of insect respiratory systems and in *Drosophila* salivary ducts [49, 50]. DIC imaging revealed the lumenal surface of the SD to be markedly smoother (Fig. 2Aiii-iv, vi) than that of the CD and ID.

A view of an entire female SG stained with labeled wheat germ agglutinin (WGA), which binds chitin and O-GlcNAcylated proteins, highlights the entire duct, consistent with the mosquito salivary duct being composed largely of chitin (Fig. 2Bi). A zoomed image of the triductal junction (TDJ) region, where the ID splits to form the individual SDs in the female gland, showed that chitin levels in the SDs (Fig. 2Bii, *yellow arrow*) are much higher than in the ID (Fig. 2Bii, *white arrow*). Images of ducts stained with WGA and DAPI illustrate the close association of nuclei with the chitinous common (CD) and individual (ID) ducts (Fig. 2Biii-iv). Only rarely were nuclei observed away from the CD (Fig. 2Biv, *white arrow*), or in tight association with the SD inside the female or male SGs (Fig. 2Cviii, *yellow arrows*). WGA staining was also seen in the cells of the SG, in the female DL and M lobe cell bodies and the secretory cavities of the PL lobe (Fig. 2c). In the male gland, WGA staining was high in the chitinous SD and lower in the secretory cavities (Fig. 2d), similar to the staining observed in the female PL lobe. The WGA staining in gland cells and secretory cavities, in combination with the relatively stronger WGA staining in the SD versus the CD and ID, suggest that chitin synthesized by gland cells contributes to the SD. How the SD forms and which cells contribute are unclear.

#### Variations in salivary gland architecture

**Fig. 3:**
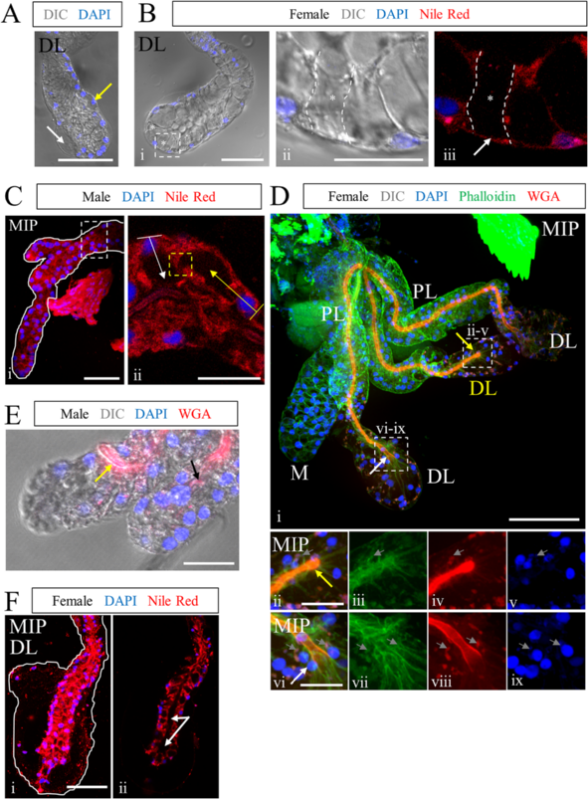
Salivary gland architecture exhibits highly variable morphological features. Images are examples of the characteristics observed and quantified in Table 4. **a** DIC imaging of a female DL lobe stained with DAPI (nuclei). Two areas of non-cellular basal ECM are visible (arrows). An isolated cell is sometimes observed associated with the basal ECM (*yellow arrow*), but not always (*white arrow*). **b** Female DL lobe stained with DAPI and Nile Red and imaged for fluorescence with DIC. SG cells were sometimes missing, as in the region outlined and marked with asterisks. **c** Male SG stained with Nile Red and DAPI. Rare instances of misoriented cells (*yellow arrow*) were observed. Typically, cells are basally positioned and apico-basal polarity (*white arrow*) is directed toward the lumen/duct. In this example, the secretory cavity runs parallel to the duct (*yellow arrow*), and opens into an adjacent secretory cavity, not the lumen. **d** Four-lobed female adult SG stained with WGA (chitin, O-GlcNAc groups), Phalloidin (actin), and DAPI (nuclei). Phalloidin staining is observed along cell membranes. WGA staining highlighted duct structure. Nuclear (DAPI) and actin (phalloidin) signal and localization were similar between lateral lobes (Dii-ix). An additional lateral lobe (yellow DL in Di) is observed (Dii-v), differing from typical lateral lobes (Dvi-ix); the duct is fused (Div), but actin and nuclear position/morphology appear normal. Small DAPI bodies (see Fig. 6) are marked by gray arrows. **e** Male SG stained with WGA (chitin) and DAPI, imaged with DIC and fluorescence. Frequent bifurcation (two distal “lobes”), premature termination (black arrow), and occasional misdirected ducts (*yellow arrow*) were observed. **f** Nile Red- and DAPI- stained female DL lobe with small lumen (Fii, *white arrows*). Even without extensive lumen expansion (*white arrows*), DL lobe cells are cup-shaped with visible secretory cavities, surrounded by a large basal ECM territory and the basement membrane. Duct images not labeled “MIP” are single Z-slices. Scale bar lengths are: 50 microns—A, Bi, Ci, Di, Fi; 20 microns—Bii, Cii, Dii, Dvi, E

Staining of many glands revealed morphological features that were not common to all glands. We described and quantified these differences (Fig. 3; Table 4). Some glands contained acellular regions, presumably filled by an extracellular matrix (Fig. 3a, *white arrow*). This feature occurred frequently in the DL and PL lobes, 49.8 % and 31.1 %, respectively (Table 4). Some basal ECM areas contained basement-membrane associated nuclei with no clear connection to other nuclei or to the lumen/duct (Fig. 3a, *yellow arrow*; Table 4). There were also regions in which a cell was missing (Fig. 3Bi, asterisks); when a row of cup-shaped SG cells lacked a nucleus and surrounding cell body staining, then continued on, we counted this as a missing cell (Fig. 3b; Table 4). In these instances, basement membrane staining was still seen (Fig. 3Biii, *white arrow*). Rarely, we observed instances of aberrant cell polarity/orientation (Fig. 3c, Table 4). In most cases, the apico-basal axis was directed from the basement membrane (basal) toward the lumen/duct (apical), perpendicular to the lumen/duct (Fig. 3Cii, *white arrow*). In several instances, the apico-basal axis of a cell was directed parallel to the lumen/duct (Fig. 3Cii, *yellow arrow*); further, the secretory cavity of the cell sometimes emptied not into the lumen, but into the secretory cavity of an adjacent cell (Fig. 3Ciii; *dashed yellow box*). Several instances (Table 4) of female glands with an extra lateral lobe were observed (Fig. 3Di, *yellow* “DL”). In contrast to the vast majority of lateral lobe ducts, the SD of all extranumerary lobes had a fused terminus (similar to a male SG), but was otherwise characteristically female (Fig. 3d, *yellow arrow*). The open and fused duct phenotypes are compared in Fig. 3Dii-ix. Often in male SGs, and more rarely in female SGs (Table 4), the salivary duct was directed away from the distal end of the gland (Fig. 3e, *yellow arrow*) or terminated prematurely, not reaching the most distal region of the gland (Fig. 3e, *black arrow*). Rare (six of 269) female DL lobes showed only minimal lumenal expansion (Fig. 3Fii, *white arrows*). In such cases, DL secretory cells were cup-shaped, not squamous like duct cells, and were surrounded by a large territory of basal ECM. In summary, *An. stephensi* SG morphology varied considerably among individual mosquitoes (regardless of sex), within lobes of the same SG, and between different regions of the same SG lobe.

**Table 4:** Frequencies of *Anopheles stephensi* salivary gland morphological characteristics

	DL	PL	M	male
Basal ECM	49.8 %	31.1 %	2.1 %	25.0 %
Basal ECM-associated cells	39.4 %	19.1 %	4.3 %	78.9 %
Missing cells	11.2 %	2.2 %	4.3 %	2.6 %
Misoriented cells	0.7 %	0.4 %	0.0 %	2.6 %
Extra lobes/bifurcations	0.4 %	0.4 %	0.0 %	97.4 %
Closed duct	4.5 %	0.0 %	0.7 %	98.7 %
Misdirected/premature duct term.	1.5 %	0.0 %	0.0 %	77.6 %
**sample size**	**269**	**225**	**141**	**76**

#### Drosophila adult salivary gland architecture

**Fig. 4:**
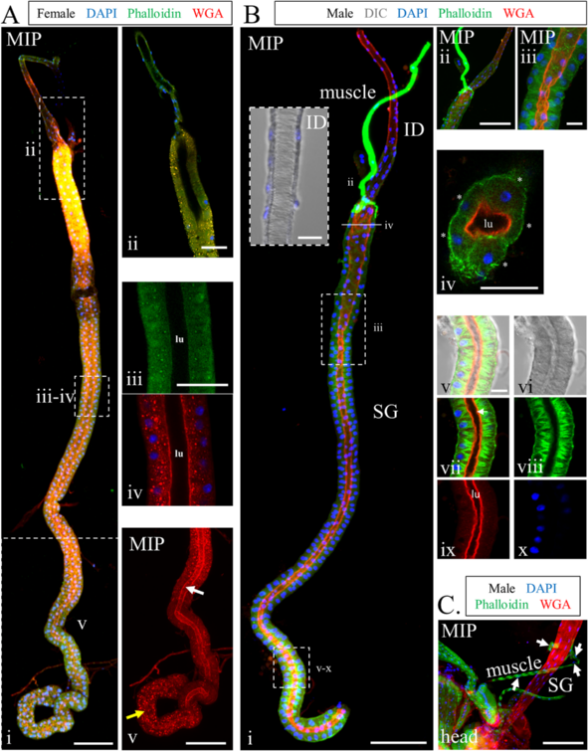
Adult salivary gland cellular architecture of *Drosophila melanogaster*. Maximum intensity projection (MIP) or single slice confocal images of female (**a**) or male (**b**-**c**) *Drosophila melanogaster* adult SGs stained with DAPI, Phalloidin, and WGA. Shown in Ai is an entire female adult SG. Cropped images are shown for the duct/proximal SG (Aii), central region (Aiii), and the distal coiled region (Aiv). Actin signal is less well organized in much of this SG (Aiii), compared to Fig. 4b. Large WGA stained granules are seen in many SG cells (Aiv), and the apical face of the cells, which defines the lumen, stains weaker and appears disorganized and/or not present in the distal coiled region (Av, compare yellow and white arrows). Bi shows a nearly complete male Drosophila SG. Nuclei are in close proximity to the duct in the ID, and taenidial rings are visible (inset). The proximal end of the SG appears to be constricted due to a muscle wrapping around it (Bii). The salivary lumen (WGA) appears wider near the proximal end (Biii). Five cells were consistently seen surrounding the lumen in cross-sections over the full length of the gland (Biv). Bv highlights the distal coiled region, where phalloidin appears enriched, and is seen in fibers running mostly apico-basally within each cell. Some cells show less actin filament organization. WGA (chitin) nicely illustrates the apical surface/lumen (Bvii, *white arrow*). In the image of a SG attached to the head (**c**), the green structure labeled muscle (Bi) was confirmed to be muscle based on the striated actin pattern and multiple nuclei not separated by cell membranes (**c**, *white arrows*). Scale bar lengths are: 50 microns—Ai, Av, Bi, Bii, C; 20 microns—Aii, Aiii, Biv; 10 microns—Bi-inset, Biii, Bv

To better understand the level of SG architectural conservation among Dipteran species, we stained and imaged adult *Drosophila melanogaster* SGs using the same set of biological dyes and the same staining protocol. Each adult gland was composed of an individual duct, with closely-associated nuclei and taenidia (Fig. 4Bi-inset), which runs from the common duct near the mouthparts all the way to the mono-lobed SGs. WGA-staining was observed at high levels on the apical cell surface in the individual duct and within the SG, with somewhat lower levels of apical staining in a region between the SG and SD, where the imaginal ring is found in the larval glands (Additional file 1: Figure S3). Inside the *Drosophila* SG, the duct and apical cell surface were coincident (Fig. 4Av, Biv, vii), lacking the periductal space observed in *Anopheles* SGs (Fig. 1Evi—“lu”). Lower level WGA staining was also observed throughout the cell bodies, sometimes in large punctate structures (Fig. 4Aiii-v, Bix). In some preparations, a striated actin-rich, multi-nucleated structure was found attached to the SG, separating the region with lower WGA staining from the rest of the secretory gland (Fig. 4Bi-ii, c). Just distal to where this structure contacts the gland, the lumen was sometimes wider for some distance into the gland (Fig. 4Biii). Enriched phalloidin signal was observed at different regions along the length of the gland in different individuals, likely correlating with age (younger—proximal enrichment; older—distal enrichment). Age also correlated with chitin differences in the distal coiled region [younger—weaker apical WGA signal (Fig. 4Av, *white arrow*) and stronger punctate WGA signal (Fig. 4Av, *yellow arrow*), and vice-versa in older SGs; Fig. 4Av, Bi]. Among multiple individuals, we find that differences in phalloidin and WGA are not sex-specific (Fig. 4, Additional file 1: Figure S2A, data not shown). Cellular actin localization varied with lumenal expansion; actin was circumferentially enriched in stretched proximal cells, whereas prominent actin fibers ran parallel to the apical-basal axis in less stretched distal cells (compare Fig. 4Biii, viii). Throughout the gland, nuclei were not as basally positioned as in mosquito adult SGs, nor were the secretory cells cup-shaped. Five approximately cuboidal shaped cells were consistently observed surrounding the lumen along nearly the entire length of the secretory portion of the gland (Fig. 4Biv). We sometimes saw evidence of cell turnover; cells were found detached from the basement membrane (Additional file 1: Figure S2A, yellow arrow) and some cells had fragmented DNA (Additional file 1: Figure S2A, white arrow). Additional features included variations in tube diameter in the region between the distal duct and proximal secretory cells, and occasional debris in the lumen (Additional file 1: Figure S2Biv, white arrow). In sum, certain major aspects of SG architecture are conserved across Dipteran species, including duct-associated nuclei, apical WGA staining of the duct and apical region of the secretory cells, and a basal bias in nuclear positioning. Other features are more distinct, including the absence of multiple lobes in female *Drosophila* SGs, the absence of branching in male Drosophila SGs, the absence of secretory cavities in Drosophila SG cells, as well as the absence of a chitinous duct separated from the secretory cells by a lumenal space in the secretory portion of the Drosophila SGs.

#### *Anopheles stephensi* salivary proteins are localized to the secretory pathway

**Fig. 5:**
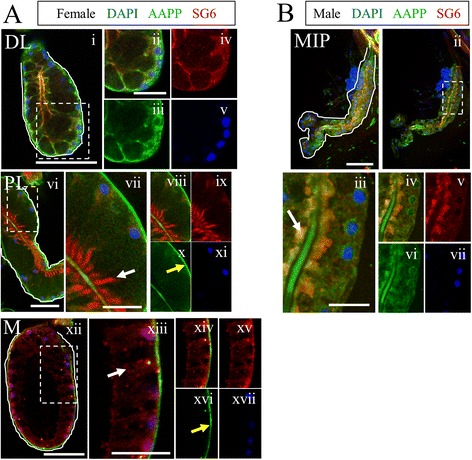
Secreted salivary protein components are localized within SG cells and secretory cavities. Confocal single slice images of adult *An. stephensi* SG lobes of females (**a**) and males (**b**) imaged by DIC (not shown; lobes are outlined thinly in *white*), stained with DAPI (nuclei, *blue*) and antibodies against the mosquito SG protein products SG6 (*red*) and AAPP (*green*). Dashed boxes indicate regions magnified in images to the right. SG6 is observed as punctate cytosolic foci in all female lobes and male SGs (e.g. Axiii, *white arrow*). Accumulation of SG6 was seen in female PL lobe secretory cavities (Avii, *arrow*). AAPP staining is seen weakly throughout the cell body in female PL and male lobes, with basolateral enrichment in the DL lobe, and basal enrichment in the PL and M lobes (Ax, xvi, *yellow arrows*). Both proteins are seen in the secretory cavities of male SGs (Biii, *arrow*) and SG6 is seen in the secretory cavity of the female PL lobe (vi-ix). AAPP signal is also observed in the duct and perinuclearly in males (Bvi). Scale bar lengths are: 50 microns—Ai, Avi, Axii, Bi; 20 microns—Aii, Avii, Axiii, Biii

We applied our immunofluorescence method to a suite of previously characterized antibodies that recognize *Anopheles* SG products (Table 1). Staining with antiserum targeting SG6, a small protein with an unknown role in blood feeding [24], was observed in punctate vesicular structures in the cell bodies in all male and female secretory cells with higher levels in the cell bodies of the female DL and M lobes [Fig. 5Aiv, xiii (*white arrow*)]. Notably, high levels of accumulation were also observed in the secretory cavities of the female PL lobe and the male SG (Fig. 5Avii, Biii; *white arrows*). The SG6 antiserum was raised against the *An. gambiae* protein; due to a high degree of homology between *An. gambiae* and *An. stephensi* homologs (Additional file 1: Figures S5 and S10; Table 3), we expect this antibody cross-reacted with *An. stephensi* SG6. Antiserum targeting AAPP, a reportedly female-specific *Anopheles* inhibitor of collagen-induced platelet aggregation [25], was observed in punctate vesicular structures in the cell bodies of the female DL, with some perinuclear enrichment likely to correspond to the Golgi (Fig. 5Aiii). Surprisingly, the male glands showed very high levels of AAPP signal in the secretory cavities, as well as lower levels of vesicular staining in the cell bodies with enrichment in the perinuclear Golgi region (Fig. 5b). Staining in male glands contradicts previous work suggesting that AAPP is female-specific [25]. In support of our findings, a recently published RNA-seq study shows expression of both SG6 and AAPP in both adult female and male *An. stephensi* [31]. We sometimes observed basal and/or duct enrichment of AAPP in the female PL and M lobes (Fig. 5Ax, xvi; *yellow arrows*), but this staining, as well as duct staining, was also observed with secondary antisera alone (Additional file 1: Figure S4C; see Methods). We applied the same staining methods with antisera to several other salivary gland and mosquito proteins (Saglin [22]; SG4 and SG5 [27]), but observed little or no signal (data not shown). Altogether, these results highlight the utility of our immunostaining protocol with a subset of available mosquito antibodies and suggest that the female PL and male gland cells are not only similar in structure but perhaps also in function.

#### Identification of a population of SG cells characterized by high nucleus to cytoplasm ratio

**Fig. 6:**
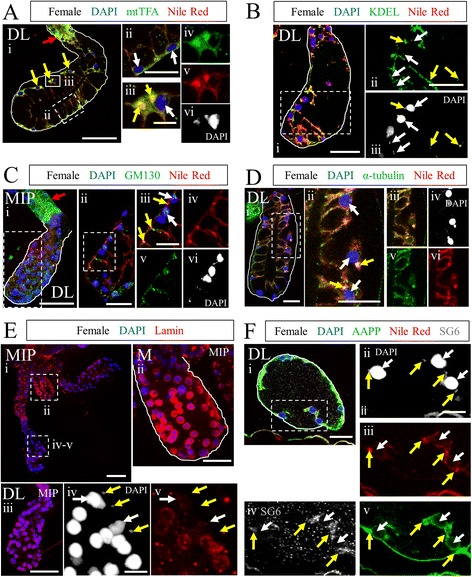
Identification of a second, smaller cell population in SGs. **a**-**f** Maximum intensity projection (MIP) images or single slices (unlabeled) of *An, stephensi* distal lateral lobes exhibiting small nuclei stained with DAPI (A-F), Nile Red (A-D, F), and: mtTFA (**a**), KDEL (**b**), GM130 (**c**), α-tubulin (**d**), lamin (**e**), or SG6 and AAPP (**f**). The small nucleus phenotype is not restricted to the DL lobe or to females (Table 4). Results in (**a**) show different cell shapes on opposing sides of this DL lobe, cup shaped (dashed box) and squamous (*yellow arrows/solid box*). The squamous cells contain multiple DAPI bodies of two size classes (large and small). Small and large nuclei are sometimes found in the same cell, but are also observed alone in separate cells (Aiv-vi, two cells: left—small nucleus; right—large nucleus and small nucleus). Small nucleus—containing cells (yellow arrows) typically have a much greater nucleus to cytoplasm ratio than large nucleus-containing secretory cells (white arrows). Small nucleus-containing cells are enriched for mtTFA (**a**), α-tubulin (**d**), and AAPP (**f**). KDEL (**b**), GM130 (**c**), and SG6 (**f**) are present, but not enriched, in small nucleus-containing cells. GM130 and mtTFA levels are high in the transition zone (Ai and Ci, *red arrows*). Lamin (nuclear envelope) is robust in the M lobe (Eii) and many DL lobes (Eiii), but is frequently disrupted or missing in secretory cells (Eiv-v, *white arrows*) in lobes where small nucleus-containing cells (Eiv-v, *yellow arrows*) are seen. **g** Scale bar lengths are: 50 microns—Ai, Bi, Ci, Ei, Eiii; 20 microns—Aii, Bii, Cii, Ciii, Di, Dii, Eii, Fi; 10 microns—Aiii, Eiv, Fii

While analysis of stained SGs was ongoing, we noticed many instances in which not all DAPI-positive bodies within a lobe were similar in size (Fig. 3Dii, vi; *grey arrows*). Small DAPI bodies (Fig. 6; *yellow arrows*) were often observed (Table 4) and associated with Nile Red membrane staining (Fig. 6, Additional file 1: Figure S3C), but the presumptive cytoplasmic volume of these cells was much smaller than that of the large nucleus-containing secretory cells (Fig. 6, *white arrows*) well known to occupy the gland. To better understand the features of small DAPI body cells and larger secretory cells in the SGs, we identified several proteins to which antibodies are commercially or communally available, including both cytoskeletal proteins and organelle markers (Table 1). Many of these antibodies were raised against orthologous *Drosophila* proteins and have a very high degree of conservation (Additional file 1: Figures S6-S9; Table 2). All have undergone rigorous testing in other systems to ensure specificity [51–55]. Staining revealed that small DAPI bodies were positive for mtTFA, a mitochondrial transcription factor (Fig. 6Aiii-iv; *yellow arrows*). Indeed, mtTFA staining was relatively intense in the small DAPI bodies compared to the adjacent large secretory cells. To rule out the possibility that these small DAPI bodies simply corresponded to mitochondrial DNA, we examined SGs stained with antibodies against additional organellar proteins. Staining was observed with both KDEL (endoplasmic reticulum; Fig. 6b) and GM130 (Golgi; Fig. 6c) antibodies in both the DAPI bodies (yellow arrows) and in the larger nearby secretory cells (white arrows). Both mtTFA and GM130 were also highly enriched in cells of the transition zone (Fig. 6Ai and Ci, *red arrows*). Both the larger secretory cells and smaller membrane-bound DAPI bodies stained with markers for actin (Fig. 3Dii-ix, *grey arrows*) and α-tubulin (Fig. 6d). In most SGs, clusters of widely variable numbers of cells showed extreme enrichment of α-tubulin signal (data not shown). Interestingly, in contrast to SG lobes where no small DAPI bodies were observed (e.g. Fig. 6Eii-iii, M and DL lobes), staining for lamin (a nuclear membrane component) was sparse and dispersed in lobe regions with small DAPI body-containing cells (Fig. 6Eiv-v, DL lobe). This suggests that nuclear envelope breakdown may accompany the presence of small DAPI body-containing cells.

To determine whether small DAPI body-containing cells are likely of the same lineage as secretory cells, we compared staining for secreted proteins within SG secretory (white arrows) and small DAPI body-containing (yellow arrows) cells (Fig. 6f). We find that SG6 and AAPP are present in the cytoplasm of cells of both morphologies, suggesting that secretory cells and small DAPI body-containing cells are likely of the same lineage.

To gain some insight into the DNA content of the small DAPI bodies, we measured and compared the diameters of adult *Anopheles stephensi* and larval *Drosophila melanogaster* SG nuclei. The ploidy of larval *Drosophila* SG cells has been well established. Larval *Drosophila* SGs (Additional file 1: Figure S3A) are composed of three cell types: imaginal cells, duct cells, and secretory cells. The imaginal cells, the precursors to the adult SG, are known to carry a diploid DNA content; duct cells are moderately polyploid, and secretory cells are highly polytene—with a copy number up to 2^8^ or more [56]. We find that diploid imaginal cell nuclei are roughly 3.5 microns in diameter, duct nuclei are about 10 microns, and secretory nuclei are around 20 microns (Additional file 1: Figure S3B). The average diameter of *Anopheles* duct cell nuclei was around 3 microns, and secretory cell nuclear diameter was around seven microns (Additional file 1: Figure S3Ci). Small nucleus morphology varied considerably (from small and round, to compact but severely elongated, to considerably decondensed), so maximal length was measured (Additional file 1: Figure S3Cii-iii). Micronucleus maximal length averaged four microns, but ranged between 0.23 and 23 microns (Additional file 1: Figure S3Ciii). Small, possibly sub-genomic, DNA fragments and decondensed DNA were observed (Additional file 1: Figure S3Ciii, red and grey boxes, respectively). The current genome size estimates of *Drosophila melanogaster* (~150 megabases, dm6) and *Anopheles stephensi* (~220 megabases, AsteS1) are reasonably similar. Direct visual comparison of *D. melanogaster* imaginal nuclei and roughly spherical *An. stephensi* micronuclei at the same scale confirm they are similar in size (Additional file 1: Figure S3D, white arrows), although they differ in size statistically (Mann-Whitney *U* test, *p* = 0.0325). In sum, these data suggest that the membrane-bound small DAPI bodies in *Anopheles* SGs are likely diploid cells containing a variety of expected organellar and structural proteins, and that the larger *An. stephensi* secretory cells are likely moderately polyploid.

## Discussion

**Fig. 7:**
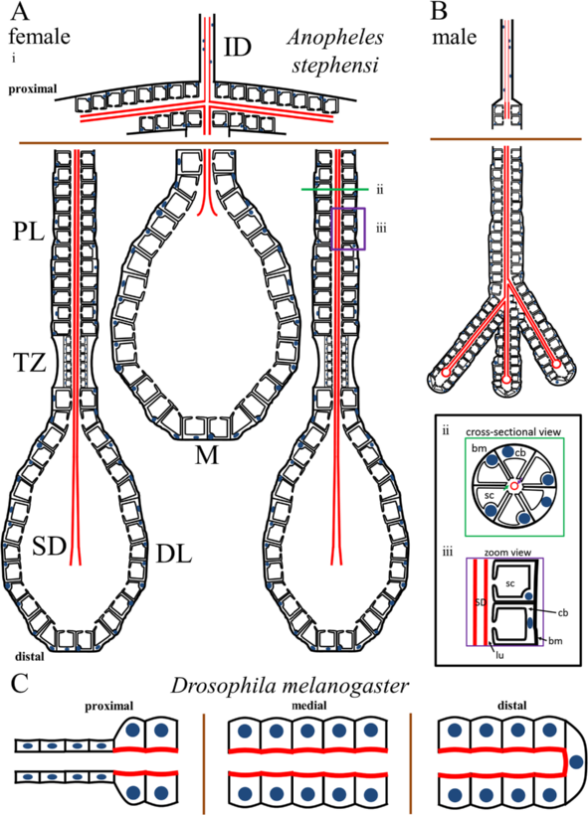
Adult Dipteran salivary gland cellular architecture. **a**-**b** Schematic diagrams of adult female (Ai) and male (**b**) *An. stephensi* SG cellular architecture. Cell shape, duct placement, and nuclear positioning are highlighted. Duct chitin (red) is more abundant inside the SG than in the individual duct. Proximal (top) and distal (bottom) are labeled. Insets: Cross-sectional (Aii) and zoom (Aiii) views of *An. stephensi* SG cells. In Aii, a purple arrow marks the SD, and a green arrow marks the lumen (lu). Individual variation in cellular architecture at many levels was documented in Fig. 3 and Table 4. **c** Schematic diagram of *D. melanogaster* SG cellular architecture. Cell shape, the cellular duct, the chitin-rich apical surface of SG cells, and the SG lumen are depicted. Note that nuclear position in *Drosophila* adult SGs is not as basally biased as in *Anopheles stephensi*. In *An. stephensi*, the chitinous duct is a separate structure within the SG, separated from secretory cells by a secretory cavity and a periductal space; in contrast, the *Drosophila* SG chitin staining is at the apical cell surface, defining the central lumen. In both species, nuclei are smaller and tightly associated with the duct in the ID, but larger and farther from the duct inside the SG. Brown lines denote segments deleted to fit the available space. Female and male *Anopheles* SGs are approximately to scale. cb: cell body, sc: secretory cavity, bm: basement membrane

We have shown that a simple, optimized fixation protocol works well for staining adult *An. stephensi* SGs with a number of biological dyes as well as antisera recognizing a diverse range of protein types: from secreted proteins, to organellar markers, to components of the cytoskeleton. Indeed, the protocol gives indistinguishable results on both freshly dissected and frozen samples (Additional file 1: Figure S4). Importantly, antisera generated against Drosophila proteins with high levels of sequence identity to their mosquito counterparts (Additional file 1: Figure S6-S9) worked quite well, indicating that Drosophila antibodies will be excellent tools for studying a wide range of processes in mosquitoes. Confocal imaging of the stained glands has allowed us to compare SG and cellular architecture among a large number of individuals, a comparison that is simply not possible with TEM. A diagram summarizing SG architecture in *Anopheles stephensi* based on our findings is shown in Fig. 7, bearing in mind that we observed considerable individual variation from this “typical” morphology. We are encouraged that most of the architectural features we observed are quite similar to those described by Wright in the late 1960s, in terms of organization, cell shape, and organelle location. We see that the organization of the male gland is quite variable, and in the majority of cases (97.6 % of glands examined), male SGs contain multiple branches and/or lobes. Accounts of male *An. stephensi* SG morphology indicate wide variability, from a single lobe to multiple lobes and multiple salivary duct branches [11]. A similarly variable, multiple-lobe male gland morphology has been described in *Anopheles albimanus* and several other mosquito species [13, 16, 57]. Morphological variation was also observed for several features of female SGs (Fig. 3; Table 4). Morphological variation in *An. stephensi* SGs could potentially be due to differences in individual gene expression, developmental age, differences in feeding history, environmental factors, and/or differences in regional effects of natural selective pressures on lobe morphology prior to laboratory colony establishment. This degree of morphological variation could indicate that structural uniformity in these SGs is not required for function.

We are encouraged that our staining procedure reveals that secreted proteins understood to function during blood feeding are found in expected locations: either within cells in locations consistent with secretory organelles, or in the secretory cavities, poised for timely release during feeding. We were surprised to discover domain differences in where secretory proteins localized, with one localization pattern in the female medial and distal lateral lobes and another in the proximal lobe and male lobe. This finding is consistent with conclusions from the TEM analysis and our morphological characterizations, showing that the female proximal lobe is more similar in morphology to the male gland than it is to other regions of the female gland. Thus, morphological distinctions may reflect functional differences in different SG domains. It is unclear why male glands express and store such high levels of proteins thought to function in blood feeding. Perhaps these proteins have additional roles in general feeding. Alternatively, the genes encoding these proteins may simply be under the same molecular genetic control as all SG secreted gene products. Indeed, our studies of the *Drosophila* embryonic SG suggest that all the organ-specific genes of the SG are under the control of the same transcriptional cassette [58].

Sexual dimorphism in adult gland morphology is observed not only in *An. stephensi*, it is broadly found across hematophagous (blood feeding) mosquito species [11, 59–63]. We see no evidence of sexual dimorphism in the adult *Drosophila* SG, although other adult structures in *Drosophila* show clear evidence of sex-specific differences, including the presence of sex combs, sexually dimorphic abdominal segment number and abdominal pigmentation patterns, and the sexually dimorphic internal and external genitalia. In these cases, dimorphism arises in two ways [64]. For some tissues, there are different primordial precursors that give rise to sexually dimorphic structures, with only one set of precursors (male or female) continuing to divide and differentiate in the two sexes. In other tissues, the same primordium is present and develops in both sexes, but the primordium follows sex-specific patterns of terminal differentiation. The *D. melanogaster* embryonic/larval SG secretory and duct cells are derived from different primordia [49, 65]. In light of this, it is interesting to consider the morphology of the extra lobes that we occasionally observed in the female glands. In all cases, the duct was closed (as in males), whereas the gland morphology appeared otherwise female (Fig. 3d). This observation might suggest that the duct arises from separate male and female primordia, whereas the SG cellular precursors may be the same in both sexes.

The origin of the chitinous duct in the secretory portion of the SG is not clear. In the common (CD) and individual ducts (ID), the chitinous taenidial structure that lines the tubes are in direct contact with the apical surface of tightly associated squamous epithelial cells, indicating that the CD and ID directly synthesize and organize duct architecture. In the glandular part of the duct, which we refer to as the SD, the secretory cells are typically separated from the chitinous duct by both the secretory cavities of individual cells and the common lumenal space surrounding the SD. Clearly, the secretory cells synthesize chitin that contributes to SD structure, but which cells form the SD template is not obvious. We propose two alternative models for the origin of the SD. In the first model, we propose that the SD is made by secretory cells that are in much closer contact with the duct when it first forms. As the secretory cells mature into the cup shaped cells of the later SG, a separation would occur between the apical surface of these cells and the duct, although the secretory cells continue to synthesize additional chitinous material to reinforce SD structure. This model is supported by the morphology of the Drosophila secretory “duct”—the chitinous lining of the secretory portion of the gland, which is in direct contact with the apical surface of the cuboidal epithelial gland cells. This idea is also supported by the rare instances where we saw glandular cells of *An. stephensi* in direct contact with the duct and lumenal space (Fig. 3f). In the second model, we propose that cells at the distal ends of the individual duct (ID cells in the triductal region) behave like the terminal cells of the Drosophila tracheal system, which send out long cytoplasm extensions which subsequently organize central internalized lumens to form the tracheoles [66, 67]. Tracheoles extend over 200 μm, consistent with size range of the SD. Like the SD of *An. stephensi*, the tracheoles produce a chitinous cuticle that lines the internalized lumens to keep the tubes open. Supporting this second model, we frequently observed Nile red (and actin) staining of the SD, suggesting that a biological membrane is associated with the SD. Also supporting this idea are the extra lobes occasionally observed in female SGs, all of which had male SD morphology and female secretory cell morphology. Tracking the origin of the SD should be feasible once our staining procedures have been adapted to staining *An. stephensi* pupal SGs.

SG morphology has been visualized during embryogenesis in both *Drosophila melanogaster* [32, 68] and *Aedes* mosquitoes [69], and in larval and adult stages in *Drosophila melanogaster* [32] and *Anopheles* species [57, 61, 70]. Embryonic SG development may occur similarly in the two species: ventral placodes of epithelial cells invaginate and collectively migrate dorsally, then posteriorly to form monolayer tubes [68, 69]. The key genetic regulators and details of embryonic SG function in mosquitoes are not well understood, whereas embryonic SG regulation and function have been well-studied in *Drosophila* embryos [68]. How the adult structure forms in mosquitoes and in flies is largely unknown. We also do not know if the same regulators of embryonic/larval gland formation play a role in the adult glands, although we would predict that they do based on gene expression profiling [17, 71]. Finally, nothing is known about the source of replacement cells for the adult SGs of flies and mosquitoes. Our studies suggest that the small DAPI bodies/micronuclei found among the mature secretory cells may be the “regenerative” cell population for the adult mosquito SG, but more work is needed to fully evaluate this possibility.

Mosquito SG morphology has been studied previously, at the tissue level by light microscopy [13–16] and the cellular level by EM [11, 12, 72]. To our knowledge, this is the first study of SG morphology at the cellular level with a large sample size. Whereas EM limits the number of salivary glands that can be analyzed, some previous light microscopy studies have involved sufficient sample size to conclude that SG lobe morphology and number can be variable in other Anopheles species [13, 16]. In contrast, a study of *Culex quinquefasciatus* SG morphology found no variability in lobe number or morphology in males or females [72]. da Cunha Sais and colleagues noted three additional differences between *Culex* SGs and those of other mosquitoes: 1) the lack of a clear non-secretory transition zone between the proximal and distal portions of the lateral lobe; 2) *Culex* PL lobe secretions were clear, whereas those of the DL and M lobes were dark in color, as previously noted for other mosquito SGs; 3) instead of an axon associated with the SGs, as seen in *Aedes aegypti* [73], *Culex* SG lobes appear to contain peripheral groups of 2–3 endocrine system-like cells [72]. We did not observe axons innervating either *An. stephensi* or *D. melanogaster* SGs; however, we did see a muscle encircling the proximal region of *D. melanogaster* SGs (Fig. 4). The diversity of overall gland morphologies and associated tissues, as well as differences in the appearance of the secretory material, coupled with the high degree of conservation at the gene level, suggests that, although Dipteran SGs are likely constructed of many of the same building blocks, the subtle differences that exist at the cellular and tissue level may impact disease transmission.

Overall salivary gland cellular architecture has important implications for parasite transmission. Female salivary ducts most often end in an open terminus (Fig. 1Aii, *arrow*), providing a direct path for parasites to enter the duct and exit the mosquito during blood feeding. We find that the female distal lateral lobe termini are fused in a non-trivial fraction of cases (4.5 %, *n* = 269; Table 4); this morphological change would certainly prevent parasite entry from those glands. The arrangement of SG cells could also impact mosquito infectivity. In some instances, SG cells barely cover one face of the basement membrane in a DL lobe, but completely fill the other face (e.g. Fig. 6Ai). Not having to traverse SG cells could allow parasites greater ease of entering the glands to access the salivary duct. Likewise, in 11.2 % of DL lobes, we observed missing SG cells (Table 4). These missing cell sites could also aid parasite entry into the SGs. Further study is required to better understand how individual SG variation affects mosquito infectivity.

## Conclusions

Overall, this study confirms many of the initial findings reported by previous authors regarding cell shape and lobe organization, and represents a step forward in terms of the kinds of *Anopheles* SG molecular biology questions that can now be addressed. With immunofluorescence, it is possible to interrogate multiple proteins thoroughly with relative ease compared to labeling and visualization by electron microscopy. Consistent immunofluorescence paves the way for other techniques we hope to adopt in the near future, including *in situ* hybridization and a genetic analysis of the factors required to generate and maintain a viable and functional SG.

## Additional file

Additional file 1:
**Figure S1.** Adult Anopheles stephensi salivary gland lobe and cellular morphometry. (A) Graph of average length (blue) and width (red) of each lobe from *An. stephensi*. Ten samples were measured per lobe. Results indicate that lobe length varies more than lobe width, but variations are similar across lobes (standard deviations are shown on the graph). (B) Graph of adult *An. stephensi* SG cellular morphometry. Each data point represents 50 cellular measurements among 10 lobes. Results show low variability in both female and male gland cellular dimensions, except for proximal lateral cell length, which varies with asymmetric positioning of the duct within that part of the gland. (C) Cross-sections from Nile Red and DAPI stained SGs illustrating the number of nuclei in circumference in the female distal lateral (DL), proximal lateral (PL), and medial lobe (M), and male gland. Proximal (P) and distal (D) are labeled on each lobe/gland for orientation. Arrow in (PL) marks the PL cross-section. The number of circumferential nuclei varies along the length of each lobe. These cell shape and duct observations agree well with previously published electron microscopy cross-sectional views [11]. Scale bars are 50 microns long. **Figure S2.** Additional details regarding adult *Drosophila* salivary gland cellular architecture. Adult *D. melanogaster* (Canton S) SGs stained with DAPI (nuclei), phalloidin (actin), and WGA (chitin; A) or Nile Red (lipids; B-D). A) Evidence of cell turnover was observed, including DNA fragmentation (white arrow), basal cell detachment, and actin accumulation (yellow arrow). (B-D) Female (B) and Male (C-D) SG cell shape. Results show Nile Red staining throughout the cell membranes, highlighting the cuboidal shape of the secretory cells (e.g. Bii, white arrows). Actin is well organized into apico-basal fibers (Biii), and debris can sometimes be visualized in the lumen (Biv, white arrow). The lumenal surface appears irregular [compare Cii to D (proximal portion from another gland)]. Images not labeled with “MIP” (maximum intensity projections), are single slices. D is a MIP of two slices. Scale bar lengths are: 50 microns—A, Bi, Ci; 20 microns—Bii, Biii, Biv, Cii, D. **Figure S3.** Larval *Drosophila* salivary gland nuclear dimension correlate with known ploidy. (A) Male *D. melanogaster* (Oregon R) larval SGs stained with DAPI (nuclei). Three classes of nuclei are visible: 1) imaginal nuclei (blue arrow), 2) duct nuclei (red arrow), and 3) secretory nuclei (green arrow). Imaginal nuclei are known to be diploid; duct nuclei are weakly polyploid and secretory nuclei are very highly polyploid (polytene). (B) Graph of nuclear diameters of these three cell types. Sample size is given at the base of each bar. Note that nuclear size increases with an increase in ploidy. (C) *An. stephensi* nuclear diameter measurements. A graph (Ci) of nuclear diameter measurements from two populations of cells (duct, and secretory cells), reveals that both cell types had consistently sized nuclei; secretory cell nuclei are a little more than twice the diameter of duct nuclei. Secretory cell and duct cell measurements were taken from Fig. 2a and similar images. Small DAPI body (micronucleus) measurements were taken from Cii. In this female DL lobe, micronuclei are abundant (sites of enriched DAPI and Nile Red colocalization). Micronucleus morphology was often elongated, not spherical, so maximal length was measured. Micronucleus length distribution is plotted in Ciii, sorted from smallest to largest. Discontinuous outlier values are boxed. (D) Visual comparison of *D. melanogaster* imaginal cell (Di) and *An. stephensi* micronucleus (Dii) size. Images are the same scale, and nuclei of interest are marked by white arrows. *: *p*-value < 0.001 by pair-wise Mann-Whitney U tests. Scale bar lengths are: 50 microns—A, Dii; 20 microns—Ci, Cii. **Figure S4.** Controls for fluorescence microscopy in *Anopheles stephensi* salivary glands. A-B) Consistency in immunofluorescence results when performed on frozen versus freshly dissected *An. stephensi* SG tissue. Shown are maximum intensity projections from either freshly dissected (A) or frozen, then thawed (B) adult female and male *An. stephensi* SGs stained with DAPI and Rh-WGA (chitin, O-GlcNAc groups). Similar staining effectiveness was observed on both tissue preparations. C) Secondary antibody background signal controls. Shown are maximum intensity projections from adult *An. stephensi* female or male SGs stained without adding primary antibody. When rabbit secondary antibodies were used in the absence of primary antibody, we detected weak duct staining and punctate cellular staining. This background signal disappeared in the presence of primary antisera against a variety of proteins (See Methods). Mouse secondary antibodies alone showed very little signal accumulation. Scale bars are 50 microns in length. **Figure S5.** Conservation of the mosquito protein AAPP. A) Multiple sequence alignment of AAPP homologs from multiple mosquito species. Green: identical residue in all proteins. Yellow: conserved residue. Teal: similar residue. B) AAPP protein conservation tree diagram. AAPP is conserved within mosquitoes, but is not present in *Drosophila*. See Methods for additional details. **Figure S6.** Conservation of the microtubule component protein α-tubulin. Multiple sequence alignment of α-tubulin homologs in mosquitoes and *Drosophila*. Green: identical residue in all proteins. Yellow: conserved residue. Teal: similar residue. **Figure S7.** Conservation of the cis-Golgi-associated protein GM130. Multiple sequence alignment of GM130 homologs in mosquitoes and *Drosophila*. Green: identical residue in all proteins. Yellow: conserved residue. Teal: similar residue. **Figure S8.** Conservation of the ER-associated KDEL receptor. Multiple sequence alignment of KDEL receptor homologs in mosquitoes and *Drosophila*. Green: identical residue in all proteins. Yellow: conserved residue. Teal: similar residue. **Figure S9.** Conservation of the mitochondrial transcription factor mtTFA. Multiple sequence alignment of mtTFA homologs in mosquitoes and *Drosophila*. Green: identical residue in all proteins. Yellow: conserved residue. Teal: similar residue. **Figure S10.** Conservation of the mosquito protein SG6. A) Multiple sequence alignment of SG6 homologs in several mosquito species. Green: identical residue in all proteins. Yellow: conserved residue. Teal: similar residue. B) SG6 protein conservation tree diagram. SG6 is conserved within mosquitoes, but is not present in *Drosophila*. See Methods for additional details. (PDF 7128 kb)

## Abbreviations

bm: Basement membrane
cb: Cell body
CD: Common (salivary) duct
D: Distal
DIC: Differential interference contrast (microscopy)
DL: Distal lateral (portion of female SG)
EM: Electron microscopy
ID: Individual (salivary) duct
lu: Lumen
MIP: Maximum intensity projection
M: Medial (female SG)
P: Proximal
PL: Proximal lateral (portion of female SG)
sc: Secretory cavity
SD: Salivary duct in region of secretory gland
SG: Salivary gland
TDJ: Triductal junction
TZ: Transition zone
WGA: Wheat germ agglutinin (dye)
