# Supplementary material for: “Salivary gland cellular architecture in the Asian malaria vector mosquito Anopheles stephensi”
Source: Parasit Vectors. 2015 Dec 2;8:617. doi: 10.1186/s13071-015-1229-z (PMC4667400; doi:10.1186/s13071-015-1229-z)

**Additional File 1. Supplementary Figures and Legends**

**Figure S1. Adult Anopheles stephensi salivary gland lobe and cellular morphometry.** (A) Graph of average length (blue) and width (red) of each lobe from *An. stephensi*. Ten samples were measured per lobe. Results indicate that lobe length varies more than lobe width, but variations are similar across lobes (standard deviations are shown on the graph). (B) Graph of adult *An. stephensi* SG cellular morphometry. Each data point represents 50 cellular measurements among 10 lobes. Results show low variability in both female and male gland cellular dimensions, except for proximal lateral cell length, which varies with asymmetric positioning of the duct within that part of the gland. (C) Cross-sections from Nile Red and DAPI stained SGs illustrating the number of nuclei in circumference in the female distal lateral (DL), proximal lateral (PL), and medial lobe (M), and male gland. Proximal (P) and distal (D) are labeled on each lobe/gland for orientation. Arrow in (PL) marks the PL cross-section. The number of circumferential nuclei varies along the length of each lobe. These cell shape and duct observations agree well with previously published electron microscopy cross-sectional views [11]. Scale bars are 50 microns long.

**Figure S2. Additional details regarding adult *Drosophila* salivary gland cellular architecture.** Adult *D. melanogaster* (Canton S) SGs stained with DAPI (nuclei), phalloidin (actin), and WGA (chitin; A) or Nile Red (lipids; B-D). A) Evidence of cell turnover was observed, including DNA fragmentation (white arrow), basal cell detachment, and actin accumulation (yellow arrow). (B-D) Female (B) and Male (C-D) SG cell shape. Results show Nile Red staining throughout the cell membranes, highlighting the cuboidal shape of the

secretory cells (e.g. Bii, white arrows). Actin is well organized into apico-basal fibers (Biii), and debris can sometimes be visualized in the lumen (Biv, white arrow). The lumenal surface appears irregular [compare Cii to D (proximal portion from another gland)]. Images not labeled with "MIP" (maximum intensity projections), are single slices. D is a MIP of two slices. Scale bar lengths are: 50 microns – A, Bi, Ci; 20 microns – Bii, Biii, Biv, Cii, D.

**Figure S3. Larval *Drosophila* salivary gland nuclear dimension correlate with known ploidy.** (A) Male *D. melanogaster* (Oregon R) larval SGs stained with DAPI (nuclei). Three classes of nuclei are visible: 1) imaginal nuclei (blue arrow), 2) duct nuclei (red arrow), and 3) secretory nuclei (green arrow). Imaginal nuclei are known to be diploid; duct nuclei are weakly polyploid and secretory nuclei are very highly polyploid (polytene). (B) Graph of nuclear diameters of these three cell types. Sample size is given at the base of each bar. Note that nuclear size increases with an increase in ploidy. (C) *An. stephensi* nuclear diameter measurements. A graph (Ci) of nuclear diameter measurements from two populations of cells (duct, and secretory cells), reveals that both cell types had consistently sized nuclei; secretory cell nuclei are a little more than twice the diameter of duct nuclei. Secretory cell and duct cell measurements were taken from Fig. 2A and similar images. Small DAPI body (micronucleus) measurements were taken from Cii. In this female DL lobe, micronuclei are abundant (sites of enriched DAPI and Nile Red colocalization). Micronucleus morphology was often elongated, not spherical, so maximal length was measured. Micronucleus length distribution is plotted in Ciii, sorted from smallest to largest. Discontinuous outlier values are boxed. (D) Visual comparison of *D. melanogaster* imaginal cell (Di) and *An. stephensi* micronucleus (Dii) size. Images are the same

scale, and nuclei of interest are marked by white arrows. *: p-value < 0.001 by pair-wise Mann-Whitney U tests. Scale bar lengths are: 50 microns – A, Dii; 20 microns – Ci, Cii.

**Figure S4. Controls for fluorescence microscopy in *Anopheles stephensi* salivary glands.** A-B) Consistency in immunofluorescence results when performed on frozen versus freshly dissected *An. stephensi* SG tissue. Shown are maximum intensity projections from either freshly dissected (A) or frozen, then thawed (B) adult female and male *An. stephensi* SGs stained with DAPI and Rh-WGA (chitin, O-GlcNAc groups). Similar staining effectiveness was observed on both tissue preparations. C) Secondary antibody background signal controls. Shown are maximum intensity projections from adult *An. stephensi* female or male SGs stained without adding primary antibody. When rabbit secondary antibodies were used in the absence of primary antibody, we detected weak duct staining and punctate cellular staining. This background signal disappeared in the presence of primary antisera against a variety of proteins (See Methods). Mouse secondary antibodies alone showed very little signal accumulation. Scale bars are 50 microns in length.

**Figure S5. Conservation of the mosquito protein AAPP.** A) Multiple sequence alignment of AAPP homologs from multiple mosquito species. Green: identical residue in all proteins. Yellow: conserved residue. Teal: similar residue. B) AAPP protein conservation tree diagram. AAPP is conserved within mosquitoes, but is not present in *Drosophila*. See Methods for additional details.

**Figure S6. Conservation of the microtubule component protein α-tubulin.** Multiple sequence alignment of α-tubulin homologs in mosquitoes and *Drosophila*. Green: identical residue in all proteins. Yellow: conserved residue. Teal: similar residue.

**Figure S7. Conservation of the cis-Golgi-associated protein GM130.** Multiple sequence alignment of GM130 homologs in mosquitoes and *Drosophila*. Green: identical residue in all proteins. Yellow: conserved residue. Teal: similar residue.

**Figure S8. Conservation of the ER-associated KDEL receptor.** Multiple sequence alignment of KDEL receptor homologs in mosquitoes and *Drosophila*. Green: identical residue in all proteins. Yellow: conserved residue. Teal: similar residue.

**Figure S9. Conservation of the mitochondrial transcription factor mtTFA.** Multiple sequence alignment of mtTFA homologs in mosquitoes and *Drosophila*. Green: identical residue in all proteins. Yellow: conserved residue. Teal: similar residue.

**Figure S10. Conservation of the mosquito protein SG6.** A) Multiple sequence alignment of SG6 homologs in several mosquito species. Green: identical residue in all proteins. Yellow: conserved residue. Teal: similar residue. B) SG6 protein conservation tree diagram. SG6 is conserved within mosquitoes, but is not present in *Drosophila*. See Methods for additional details.

Figure S1

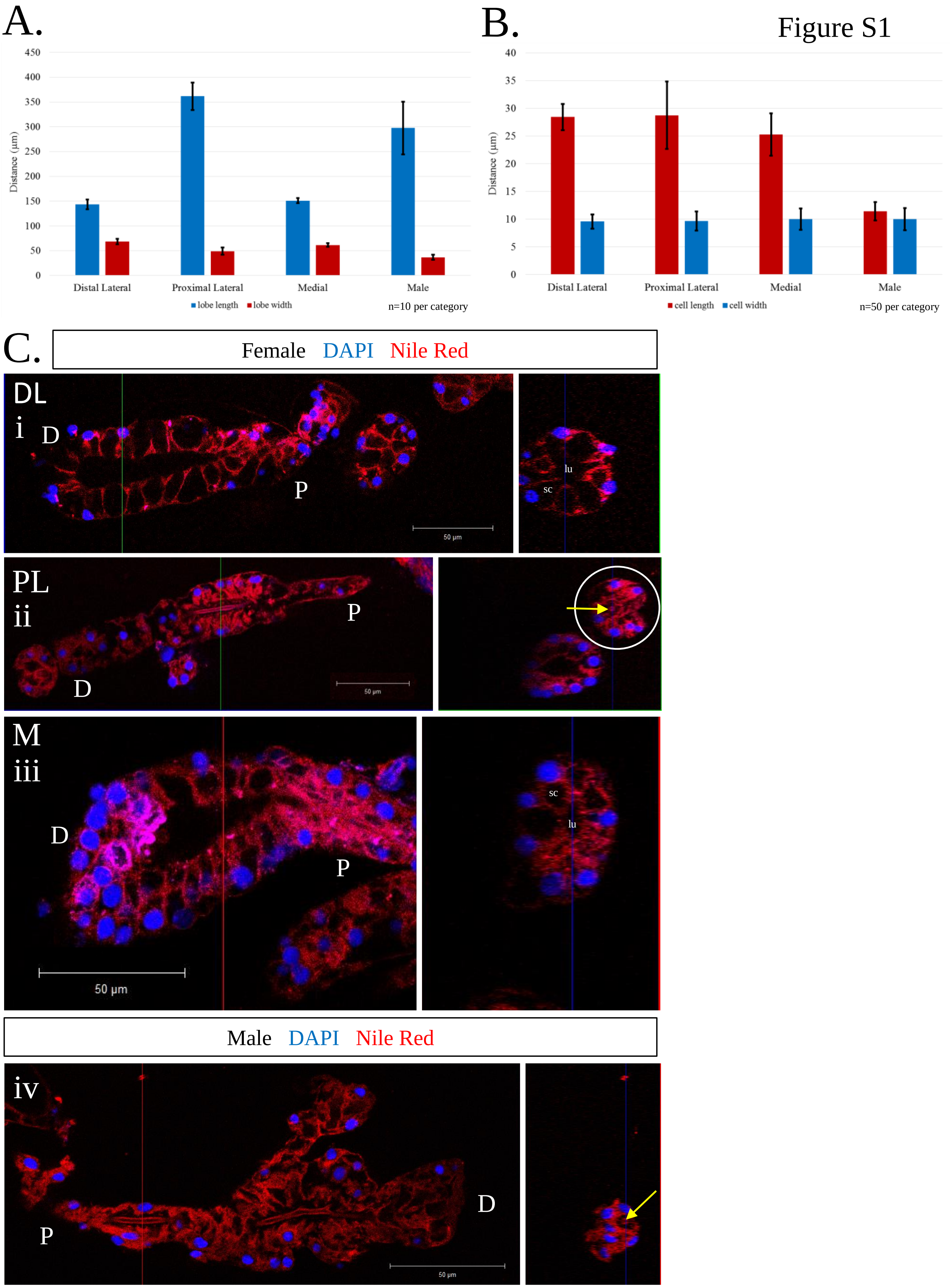

A.
450
400
350
300
250
200
150
100
50
0
Distance (µm)
Distal Lateral
Proximal Lateral
Medial
Male
lobe length
lobe width
n=10 per category
B.
40
35
30
25
20
15
10
5
0
Distance (µm)
Distal Lateral
Proximal Lateral
Medial
Male
cell length
cell width
n=50 per category
C.
Female DAPI Nile Red
DL
i
D
P
50 µm
lu
sc
PL
ii
P
D
50 µm
M
iii
D
P
50 µm
sc
lu
Male DAPI Nile Red
iv
P
D
50 µm

Figure S2

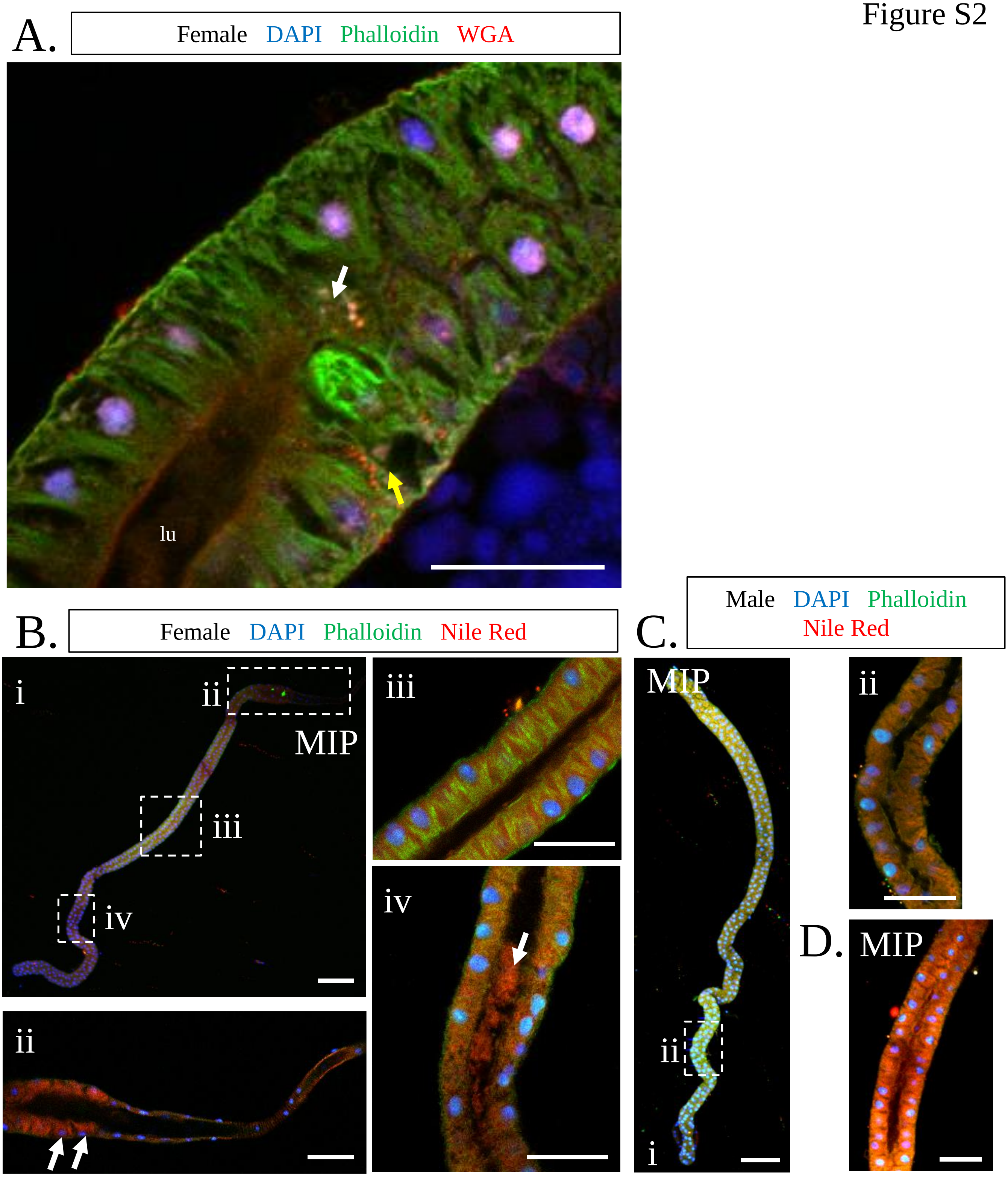
A.
Female DAPI Phalloidin WGA
lu
B.
Female DAPI Phalloidin Nile Red
i
ii
MIP
iii
iv
ii
iii
iv
C.
Male DAPI Phalloidin
Nile Red
MIP
ii
i
ii
D.
MIP

Figure S3

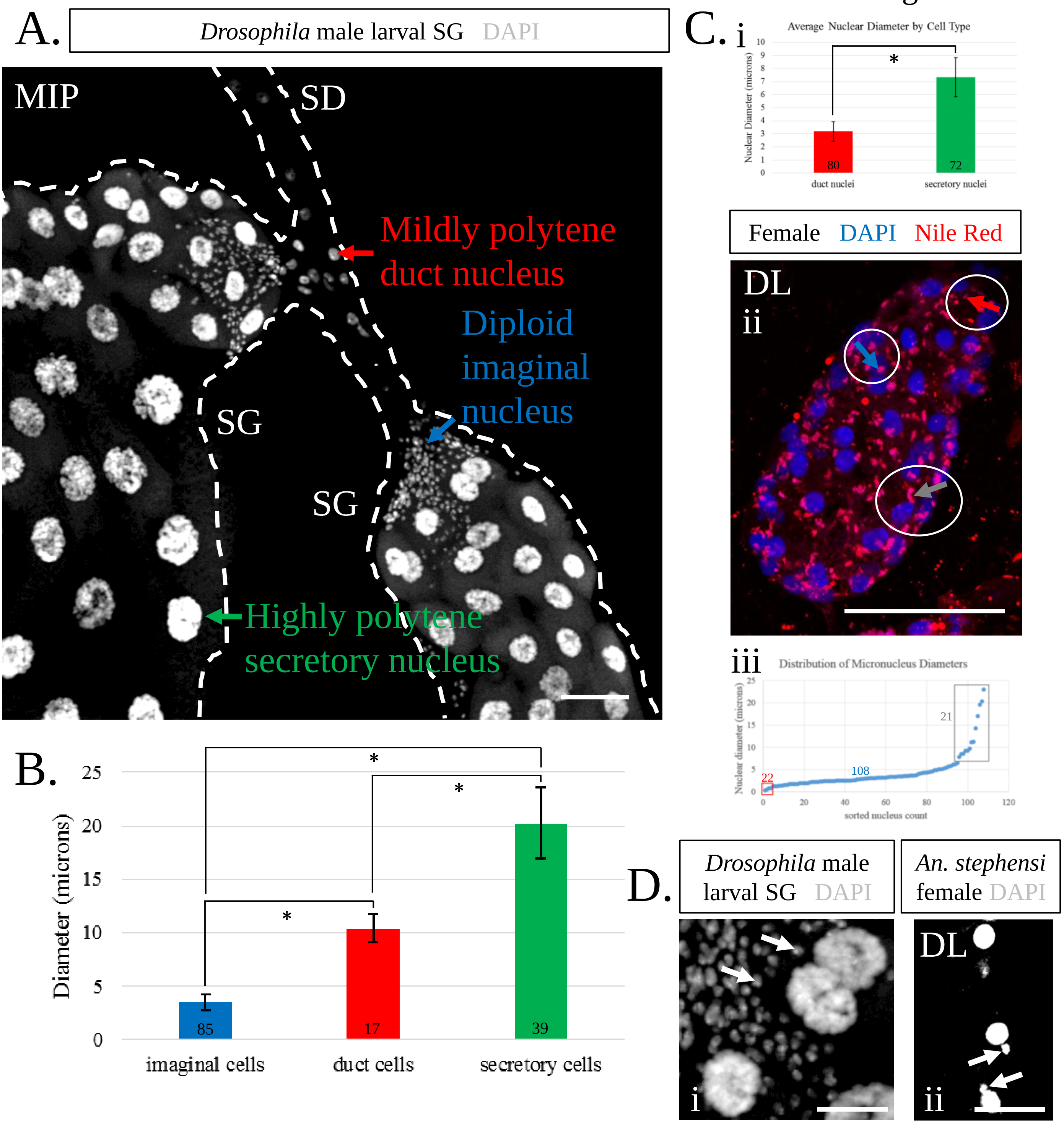
A.
Drosophila male larval SG DAPI
MIP
SD
Mildly polytene duct nucleus
Diploid imaginal nucleus
SG
SG
Highly polytene secretory nucleus
B.
Diameter (microns)
25
20
15
10
5
0
*
*
*
85
17
39
imaginal cells
duct cells
secretory cells
C. i
Average Nuclear Diameter by Cell Type
Nuclear Diameter (microns)
*
80
72
duct nuclei
secretory nuclei
Female DAPI Nile Red
DL
ii
iii
Distribution of Micronucleus Diameters
Nuclear diameter (microns)
21
108
22
sorted nucleus count
D.
Drosophila male larval SG DAPI
An. stephensi female DAPI
DL
i
ii

Figure S4

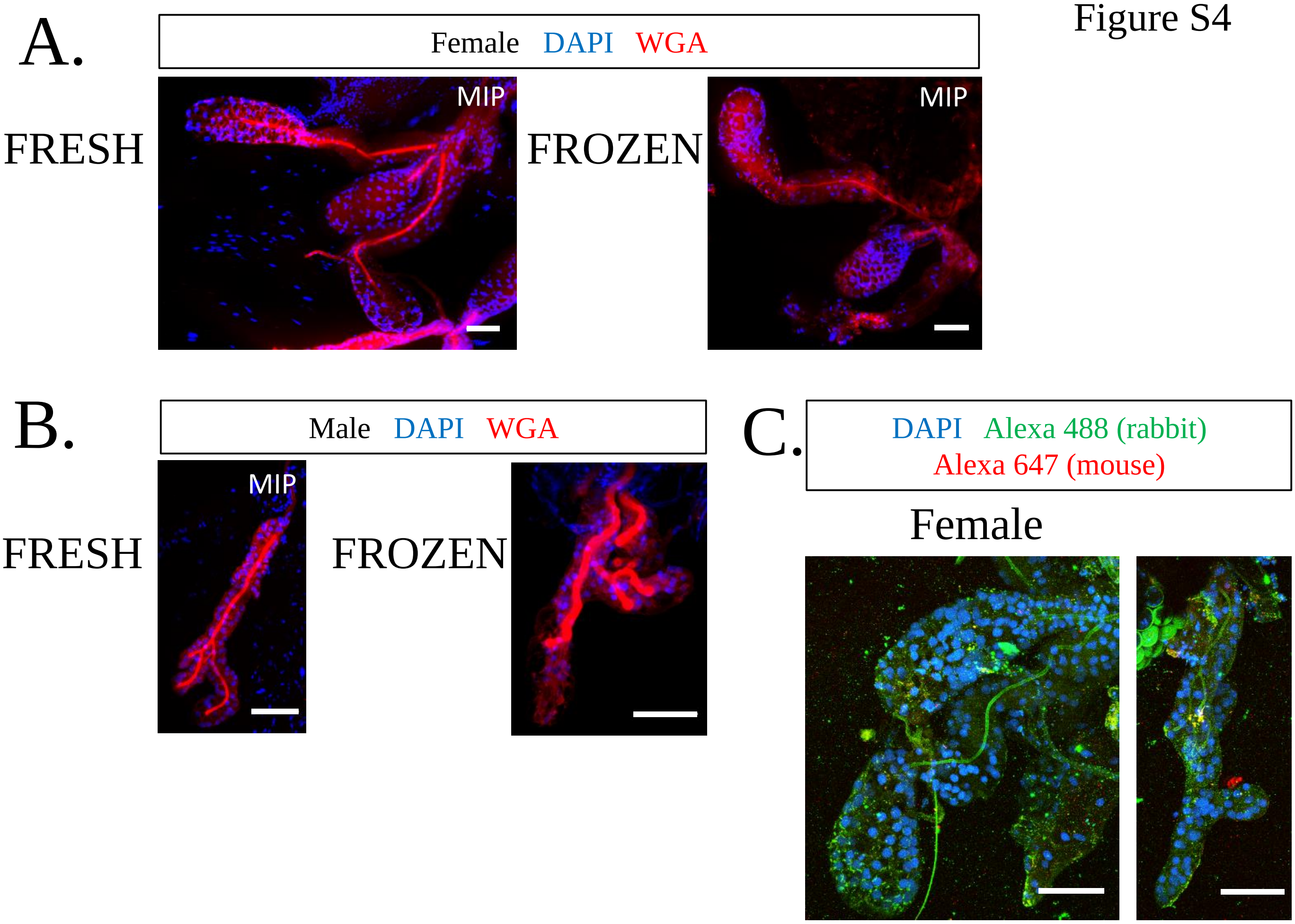
A.
Female DAPI WGA
FRESH
MIP
FROZEN
MIP
B.
Male DAPI WGA
FRESH
MIP
FROZEN
C.
DAPI Alexa 488 (rabbit)
Alexa 647 (mouse)
Female

# A.

Figure S5

```
ADAC001757 MKFYLLLASTLCLALIVTARPNEDTTEDGSSDTTGDEDG------DPSDDPRGDTPEDTTDAGDADT-----SDGHDLLTSD-------------DEVEGYGGESGDGDGTAEGEDDAES
AALB000058 MKLYLLLVSTLCLALIVAARPNEDTTEETS--ALGPEDG------DASDAPRGDTPEDTTDEGDADT-----SEEHDMLTSD-------------DEVEDHGKESENGDDTADGTDEAES
AQUA009087 MKFLLLVASVLCLVLIVSARPADDTSDQESSTELSEDAG----AEEGAEDAGSDAEADAGAADGEEG---ATDTESGAEGDD-------------SEMDSAMKEGEEGAGSDDAVSGADD
AGAP009974 MKFLLLVASVLCLVLIVSARPADDTSDQESSTELSEDAG----AEEGAEDAGSDAEADAGAADGEEG---ATDTESGAEGDD-------------SEMDSAMKEGEEGAGSDDAVSGADD
AARA003801 MKFLLLVASVLCLVLIVSARPADDTSDQESSTELSEDAG----AEEGAEDAGSDAEADAGAADGEEG---ATDTDSGAEGDD-------------SEMDSAMKEGEEGEGSDDAVSGADD
AFUN010733 MKSLLLLASVLCLALIVSARPSDETPDQETSTELSEDTSADDTQEEETVDTGAESDADSESGADAESGGDASETEDGTGESE-------------ASPDEEHTDEKASDDGEGTDDTKDE
ASTE004273 MKLLLLLASVLCLALIVSARPSDETTDQESSTELSEDTSDSYHQEEDTSEAGADAGTEGSDSEDDSSELESSSEEGHEDGSE-------------DATGEEGGAGEEGEAGEEGEAGEEG
ADIR009422 MKFLLLLAGVLCLALIVTARPQDESAD-ETTTQLSEDASEEGTHEEGESEEESDSEAGGSKGDEEGT---EGGEEDEVSDSH-------------DGPDEE-EEHSEGDDAGGDDTSEDA
AAEL010228 MKYLLTFLMALSLVNLMLTRPTPEDDGGTSE------------EPQTQET-----------------------TGSDEKN------------------GASEEPNADDASKPDDVEE
AAEL010231 -------------MIVVVLTK---------VA--------------ETTTNEA----------------------TGEITTT-------------------AASEKP-------------
AAEL010235 MKPLVKLFLLFCLVGIVLSRPMPEDEEPVAEGGDDDASGESEGEEETTDDAGGDGGEEENEGEEHAGDKDAGGEDTGKEENTGHDDAGEEDAGEEDAGEEDAGEEDAEKEEGEKE
consensus  mkfllllasvlclvlivtarp-ddttd-esst-lsed-g-----ee-sddag-d---d---add-d---------esg-e-sd-----------------e--g-e-edgd---eg----ed

ADAC001757 ADGEG------GS-------GEGGKGGAGGKE--SRRNTYRQVHKLLKKVMKVD-VKDKYLKSFIVARLQERLMNPTIDLVSTIEKYSKIKECFSSVEKDTKELVKESEKSYKECA--KD
AALB000058 TDGESDGSAEDGA-------GGSGEGGKGGKE--SARNTYRQVHKLLKKVMKVD-VKDKYLKSFIVARLQERLMNPTIDLVGNIEKYSKIKECFSSVEKDTKDLVKESEKAYKECS--ED
AQUA009087 ATEESKDDAEEGSEEGGEEGGDGASGGEGGEK-ESPRNTYRQVHKLLKKIMKVD-TKDKYLKSFVVGRLQERLMNPTIDLVSTIEKYSKIKECFSSLDKDVSAMVKESEKSYEECS--KD
AGAP009974 ETEESKDDAEEDSEEGGEEGGDGASGGEGGEK-ESPRNTYRQVHKLLKKIMKVD-TKDKYLKSFVVGRLQERLMNPTIDLVSTIEKYSKIKECFSSLDKDVSAMVKESEKSYEECS--KD
AARA003801 ETEESKDDAEEGSE----EGGDGESGGEGGEK-ESPRNTYRQVHKLLKKIMKVD-TKDKYLKSFVVGRLQERLMNPTIDLVSTIEKYSKIKECFSSLDKDVSAMVKESEKSYEECS--KD
AFUN010733 DTEGHDDASEEGAD--GEGTGEDQQGGGEGGE-ESPRNTYRQVHKLLKNIMKVG-TKDKYLKSFVLARLQERLMNPTIDLISSVQKYSKIKECFSSLAKDVGSLVKESDKSYEECS--KD
ASTE004273 EAGEEGEAGEEGGA--GEEGGAGEEGGAEGGE-ESPVNTYHQVHNLLKNIMNVG-TKNNYLKSFILARLQERLMNPTIDLVGSISKYSKIKECFDSLADDVKSLVEKSETSYEECS--KD
ADIR009422 EEGEGGDDGESDDSAEGDDKSDAGAGGKGGEEKDDPRNTYRQVHDQLKKIMKVG-TKDGYLKSFVVARLQERLMNPTIDLIGTIGKYSKIKECFSSLANDVAALVKGSEKSYEECT--KD
AAEL010228 KGDDDTAKKEDDGESKDG-EGSEKSDKEKGEPKNDPRETYNKVIEQLDQ-IKVDNVEDGHERSELAADIQRYLRNPIVDVIGSAGDFSKIAKCFKSMVGDAKKAIEEDVKGFKECTAKKD
AAEL010231 ----------------G-ECSGKHDR---------RSTCNKVNEMLNKNTDVHKIDNAFLRTFLDNNLQSHLRVPVADAIGFISNFSKIACCFTSMGPEVKKIVDEKLKSFTSCKSSKD
AAEL010235 DAGDDAGSDDGEEDSTGGDEGEDNAEDSKGSEKNDPADTYRQVVALLDKDTKVDHIQSEYLRSALNNDLQSEVRVPVVEAIGRIGDYSKIQGCFKSMGKDVKKVISEEEKKFKSCMSKKK
consensus  e--e--d-aedg-e--g-e-geg--gg-gg-e-esprnTyrqVhklLkkimkVd-tkdkylksfivarlQerlmnPtidlvgti-kySKIkeCFsSl-kdvk-lvkeseksyeeCs--kd

ADAC001757 KRNTHCGSEGTRDLDEGLIDLEQELSDCIVEKRDEDSE
AALB000058 KSNTSCGSEGTRELDDGLIDLEQELSDCIVEKRDADSE
AQUA009087 KTNTSCGTEGTRELDDGLIEREQELSDCIVDKRDAE--
AGAP009974 KTNTSCGTEGTRELDDGLIEREQELSDCIVDKRDAE--
AARA003801 KTNTSCGTEGTRELDDGLIEREQELSDCIVDKRDAE--
AFUN010733 KSNPNCGSEGTRELDEGLIEREQELSDCIVEKRDSE--
ASTE004273 KNNPHCGSEGTRELDEGLIEREQKLSDCIVEKRDSE--
ADIR009422 KTNPSCGSEGTHDLDEGLVDRQQTLSDCIVEKRDAQ--
AAEL010228 SNAYQCSQD-RSTVQDKIAKMSSKIASCVASNRS----
AAEL010231 STDSKCSQD--SSTGGQFDQITSKIKECVSSKAKGNK-
AAEL010235 S-EYQCSEDSFAAAKSKLSPITSKIKSCVSSKGR----
consensus  ksnt-Cgsegtreldeglidreqelsdcivekrdad--
```

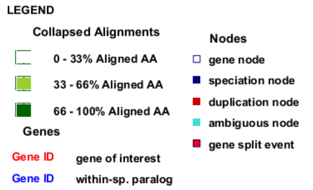


# B.

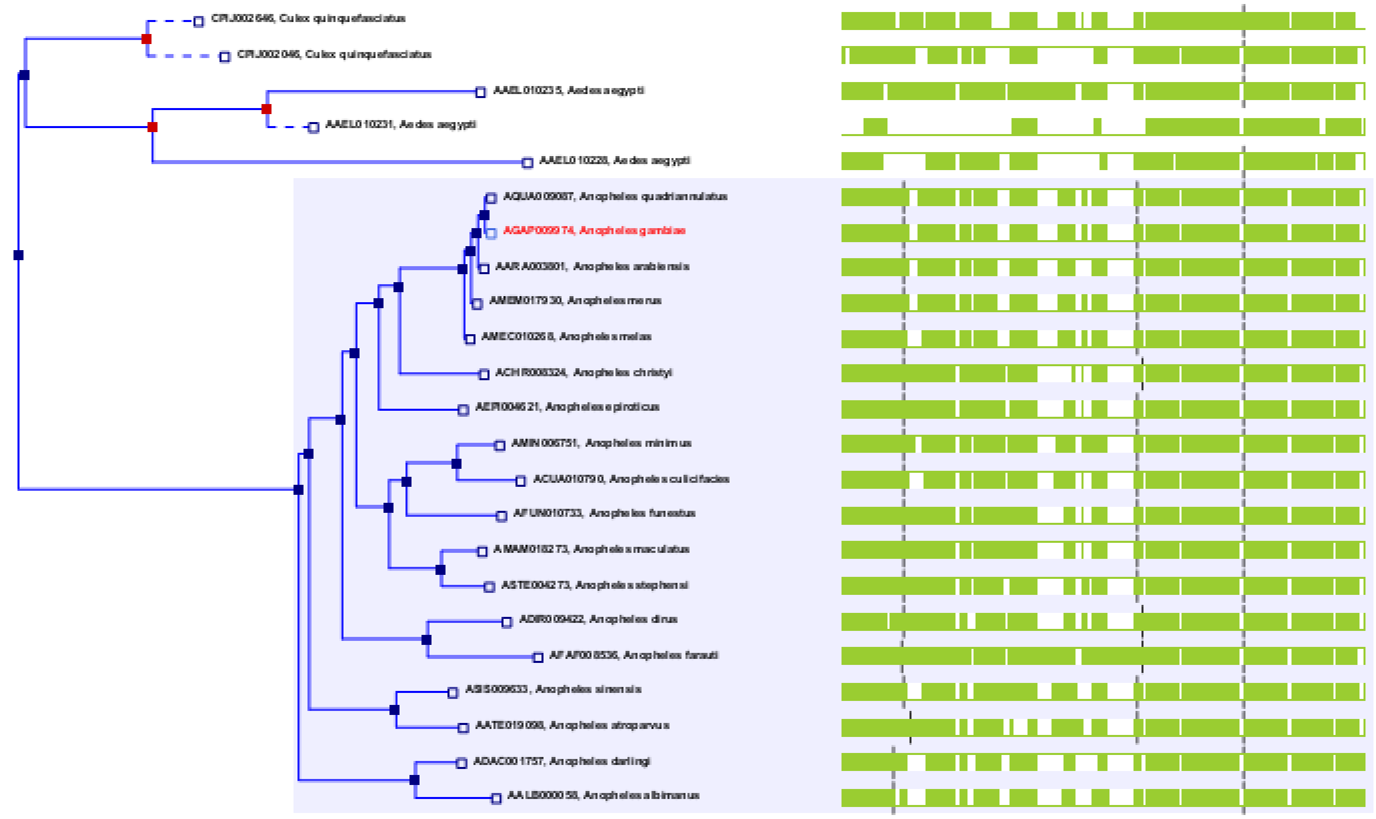

Figure S6

```
ADAC009605    MRECISVHVGQAGVQIGNACWELYCLEHGIQPDGQMPSDKTIGGGDDSFNTFFSETGAGKHVPRAVFVDLEPTVVDEVRTGTYRQLFHPEQLITGKEDAANNYARGHYTIGKEIVDIVLD
AQUA001217    MRECISVHVGQAGVQIGNACWELYCLEHGIQPDGQMPSDKTIGGGDDSFNTFFSETGAGKHVPRAVFVDLEPTVVDEVRTGTYRQLFHPEQLITGKEDAANNYARGHYTIGKEIVDLVLD
AAEL006642    MRECISVHVGQAGVQIGNACWELYCLEHGIQPDGQMPSDKTIGGGDDSFNTFFSETGAGKHVPRAVFVDLEPTVVDEVRTGTYRQLFHPEQLITGKEDAANNYARGHYTIGKEIVDVVLD
AGAP001219    MRECISVHVGQAGVQIGNACWELYCLEHGIQPDGQMPSDKTIGGGDDSFNTFFSETGAGKHVPRAVFVDLEPTVVDEVRTGTYRQLFHPEQLITGKEDAANNYARGHYTIGKEIVDVVLD
ASTE006004    MRECISVHVGQAGVQIGNACWELYCLEHGIQPDGQMPSDKTIGGGDDSFNTFFSETGAGKHVPRAVFVDLEPTVVDEVRTGTYRQLFHPEQLITGKEDAANNYARGHYTIGKEIVDVVLD
FBgn0003886   MRECISVHIGQAGVQIGNACWELYCLEHGIQPDGHMPSDKTVGGGDDSFSTFFSETGAGKHVPRAVFVDLEPTVVDEVRTGTYRQLFHPEQLITGKEDAANNYARGHYTIGKEIVDVVLD
consensus     MRECISVHvGQAGVQIGNACWELYCLEHGIQPDGqMPSDKTiGGGDDSFnTFFSETGAGKHVPRAVFVDLEPTVVDEVRTGTYRQLFHPEQLITGKEDAANNYARGHYTIGKEIVDvVLD

ADAC009605    RIRKLADQCTGLQGFLIFHSFGGGTGSGFTSLLMERLSVDYGKKSKLEFAIYPAPQVSTAVVEPYNSILTTHTTLEHSDCAFMVDNEAIYDICRRNLDIERPTYTNLNRLIGQIVSSITA
AQUA001217    RIRKLADQCTGLQGFLIFHSFGGGTGSGFTSLLMERLSVDYGKKSKLEFAIYPAPQVSTAVVEPYNSILTTHTTLEHSDCAFMVDNEAIYDICRRNLDIERPTYTNLNRLIGQIVSSITA
AAEL006642    RIRKLADQCTGLQGFLIFHSFGGGTGSGFTSLLMERLSVDYGKKSKLEFAIYPAPQVSTAVVEPYNSILTTHTTLEHSDCAFMVDNEAIYDICRRNLDIERPTYTNLNRLIGQIVSSITA
AGAP001219    RIRKLADQCTGLQGFLIFHSFGGGTGSGFTSLLMERLSVDYGKKSKLEFAIYPAPQVSTAVVEPYNSILTTHTTLEHSDCAFMVDNEAIYDICRRNLDIERPTYTNLNRLIGQIVSSITA
ASTE006004    RIRKLADQCTGLQGFLIFHSFGGGTGSGFTSLLMERLSVDYGKKSKLEFAIYPAPQVSTAVVEPYNSILTTHTTLEHSDCAFMVDNEAIYDICRRNLDIERPTYTNLNRLIGQIVSSITA
FBgn0003886   RIRKLADQCTGLQGFLVFHSFGGGTGSGFTSLLMERLSVDYGKKSKLEFSIYPAPQVSTAVVEPYNSILTTHTTLEHSDCAFMVDNEAIYDICRRNLDIERPTYMNLNRLIGQIVSSITA
consensus     RIRKLADQCTGLQGFLiFHSFGGGTGSGFTSLLMERLSVDYGKKSKLEFaIYPAPQVSTAVVEPYNSILTTHTTLEHSDCAFMVDNEAIYDICRRNLDIERPTYtNLNRLIGQIVSSITA

ADAC009605    SLRFDGALNVDLTEFQTNLVPYPRIHFPLVTYAPVISAEKAYHEQLSVAEITNACFEPANQMVKCDPRHGKYMACCMLYRGDVVPKDVNAAIATIKTKRTIQFVDWCPTGFKVGINYQPP
AQUA001217    SLRFDGALNVDLTEFQTNLVPYPRIHFPLVTYAPVISAEKAYHEQLSVSEITNACFEPANQMVKCDPRHGKYMACCMLYRGDVVPKDVNAAIATIKTKRTIQFVDWCPTGFKVGINYQPP
AAEL006642    SLRFDGALNVDLTEFQTNLVPYPRIHFPLVTYAPVISAEKAYHEQLSVAEITNACFEPANQMVKCDPRHGKYMACCMLYRGDVVPKDVNAAIATIKTKRTIQFVDWCPTGFKVGINYQPP
AGAP001219    SLRFDGALNVDLTEFQTNLVPYPRIHFPLVTYAPVISAEKAYHEQLSVAEITNACFEPANQMVKCDPRHGKYMACCMLYRGDVVPKDVNAAIATIKTKRTIQFVDWCPTGFKVGINYQPP
ASTE006004    SLRFDGALNVDLTEFQTNLVPYPRIHFPLVTYAPVISAEKAYHEQLSVAEITNACFEPANQMVKCDPRHGKYMACCMLYRGDVVPKDVNAAIATIKTKRTIQFVDWCPTGFKVGINYQPP
FBgn0003886   SLRFDGALNVDLTEFQTNLVPYPRIHFPLATYAPVISVEKAYHEQLTVAEITNACFEPANQMVKCDPRRGKYMACCMLYRGDVVPKDVNAAIATIKTKRSIQFVDWCPTGFKVGINYQPP
consensus     SLRFDGALNVDLTEFQTNLVPYPRIHFPLvTYAPVISaEKAYHEQLsVaEITNACFEPANQMVKCDPRhGKYMACCMLYRGDVVPKDVNAAIATIKTKRtIQFVDWCPTGFKVGINYQPP

ADAC009605    TVVPGGDLAKVQRAVCMLSNTTAIAEAWARLDHKFDLMYAKRAFVHWYVGEGMEEGEFSEAREDLAALEKDYEEVGMDSGEGEGEGAEEY
AQUA001217    TVVPGGDLAKVQRAVCMLSNTTAIAEAWARLDHKFDLMYAKRAFVHWYVGEGMEEGEFSEAREDLAALEKDYEEVGMDSGDGEGEGAEEY
AAEL006642    TVVPGGDLAKVQRAVCMLSNTTAIAEAWARLDHKFDLMYAKRAFVHWYVGEGMEEGEFSEAREDLAALEKDYEEVGMDSGEGEGEGAEEY
AGAP001219    TVVPGGDLAKVQRAVCMLSNTTAIAEAWARLDHKFDLMYAKRAFVHWYVGEGMEEGEFSEAREDLAALEKDYEEVGMDSGEGEGEGAEEY
ASTE006004    TVVPGGDLAKVQRAVCMLSNTTAIAEAWARLDHKFDLMYAKRAFVHWYVGEGMEEGEFSEAREDLAALEKDYEEVGMDSGEGEGEGAEEY
FBgn0003886   TVVPGGDLAKVQRAVCMLSNTTAIAEAWARLDHKFDLMYAKRAFVHWYVGEGMEEGEFAEAREDLAALEKDYEEVGIDSTTELGE-DEEY
consensus     TVVPGGDLAKVQRAVCMLSNTTAIAEAWARLDHKFDLMYAKRAFVHWYVGEGMEEGEFsEAREDLAALEKDYEEVGmDSgegeGEgaEEY
```

Figure S7

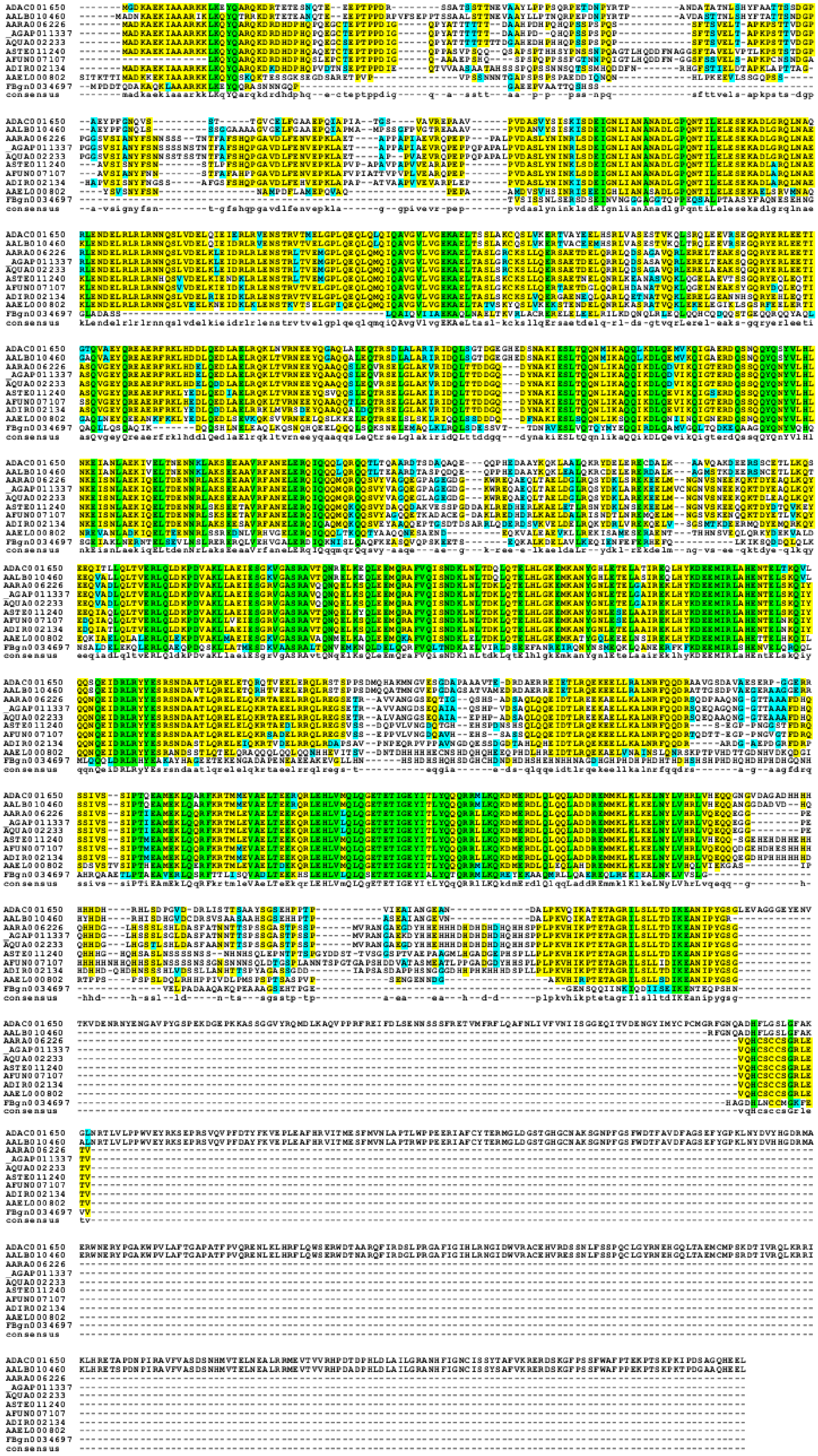

# Figure S8

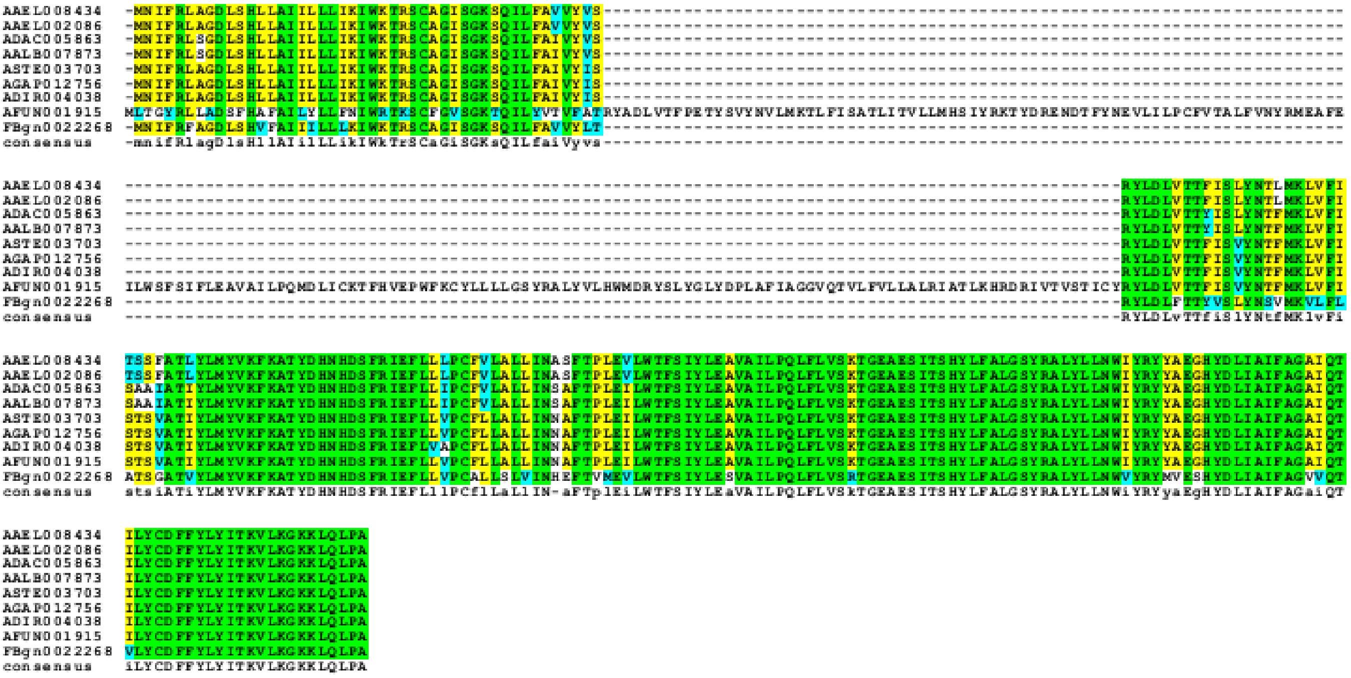
AAEL008434
AAEL002086
ADAC005863
AALB007873
ASTE003703
AGAP012756
ADIR004038
AFUN001915
FBgn0022268
consensus

# Figure S9

```
AAEL014794   MQVNNLVRLFNSSRLRISSRFLPFPRTSQPSYQLAAQFKTTSHLCDPAATSGSALPEKPKRPVNAYIRFLQSVRPSLLAKNPKASPTDISKLAAAQWQVLDPASKSKLEDEYKKEQTVWL
AAEL013643   MQVNNLVRLFNSSRLRISS------RTSLPSYQLAAQFQTTSHLSDPAATSGSALPEKPKRPVNAYIRFLQSVRPSLLAKNPKASPTDISKLAAAQWQVLDPASKSKLEDEYKKEQTVWL
ADAC006750   MQLNSLIKLFNVPRLLLSSRQSNLPLATVATQ--SCGFHSTVPLEN-AAGK-ASAPQKPKRPVNAYIRYAQSIRADLAKANPSASQMDLAKLTSAKWQTLDPPSKARLEEEYKRELAVWL
AALB000722   MQLNSLIKLFNVPRLLLSSRQNNLPLAAVATQ--SNGFHSTVPLEN-ATAK-AAAMQKPKRPVNAYIRYAQSIRADLAKANPSASQMDLAKLTSAKWQTLDQPSKARLEEEYKRELAVWL
AARA011078   MQLNAVARLFYSPRLLLSTRQPGS-LSSVPS----CGLHATATLND-AGTATKSVPEKPKRPMNTYIRFAQSIRSSLASANPQASPTDISKLAAVKWQSLDQATKAKLEEEYKREQAVWL
AGAP008499   MQLNAVARLFYSPRLLLSTRQPGS-LSSVPS----CGLHATATLND-AGTATKSVPEKPKRPMNTYIRFAQSIRSSLASANPQASPTDISKLAAVKWQSLDQATKAKLEEEYKREQAVWL
AQUA003532   MQLNAVARLFYSPRLLLSTRQPGS-LSSVPS----CGLHATATLND-AGTATKSVPEKPKRPMNTYIRFAQSIRSSLASANPQASPTDISKLAAVKWQSLDQASKAKLEEEYKREQAVWL
ASTE007783   MQLNTMARLLYSPRLLLSSWQTGP-LASAPT----AGLHCTTSLNV-A----AGTPVKPKRPMNAYIRFAQGIRSSLASANPKASPTDISKLAAVQWQSLDQASKAKYEEEYKREQAVWL
AFUN003837   ---------------------------------------------------------------MNAYIRYAQGIRSSLVSANPKASPTEISKLAAVKWQSLDQASKTKYEEEFKREQAVWL
ADIR004850   MQFNAMARFLCSSRLLLSTRQTSTPFAAVQI----SGLHSTTCLNV-DGTS-KAMPEKPKRPVNAYIRFAQSIRSSLATSNPKASPTEISKLAAVKWQTLDQASKTKLEEEFKREQAVWL
FBgn0038805  --MIYTTTLMSSRGGLIGSLINKVRPLAAAS------ISNTPAVPSKTLEEQLGLPPRPKKPLTPYFRFMREQRPKLKAANPQITTVEVVRQLSKNWSDADAQLKERLQAEFKRDQQIYV
consensus    mqln-l-rlf-sprlllssrq----l-svps-----glh-t--l---aat---alpekpkrpvnaYiRfaqsiRssLasaNP-asptdisklaavkWqtlDqasKakleeEyKreqavwl

AAEL014794   QKNAKYLSQLTDQQKQDIRQARTEKTEEKAKREYKKKVKELGRPKRPLNGFLLYCADKKP-K-NLSKEENKLQLKLMAQKWAALSDMDKAPYNTRAAEALVKYREDIKNWEDKMIAQDHL
AAEL013643   QKNAKYLSQLTDQQKQDIRQARTEKTEEKAKREYKKKVKELGRPKRPLNGFLLYCADKKP-K-NLSKEENKLQLKLMAQKWAALSEMDKAPYNTRAAEALVKYREDIKNWEDQMIAQDHL
ADAC006750   QQNAKYLSQLTEAQKEELKQDRQQKADDKAKRDLKRTLKQLGRPKRPINGFLRFCAQYKP-KPGVTQGEHKEQMKALGEKWRAMSESEKERFNREAEQAITQYQEEMKQWEDKMLAMDNG
AALB000722   QQNAKYLSQLTDVQKEELKQDRQQKAEDKAKRDLKRTLKQLGRPKRPINGFLRFCAQFKP-KSGVTQGEHKEQMRALGEKWRSMSASEKEQFNREADEAIARYQEEMKQWEDKMLAMDNG
AARA011078   QKNAKYLSQLTDAQKAEIKLERQQRNEGKVKREQKRMLKELGRPKRPMNAYLRFCAQNKP-APGLSKEDNKMQMKNLGMQWKRLPEGERERYTKEAEAEMKRYQEEMKVWEDKMLAAENV
AGAP008499   QKNAKYLSQLTDAQKAEIKLERQQRNEGKVKREQKRMLKELGRPKRPMNAYLRFCAQNKP-APGLSKEDNKMQMKNLGMQWKRLPEGERERYTKEAEAEMKRYQEEMKVWEDKMLAAENV
AQUA003532   QKNAKYLSQLTDAQKEEIKLERQQRNEGKVKREQKRMLKELGRPKRPMNAYLRFCAQNKP-APGLSKEDNKMQMKNLGMQWKRLPEGERERYTKEAEAEMKRYQEEMKVWEDKMLAAENV
ASTE007783   QKNAKYLSQLTDAQKEEIKLERQQRNEGKAKREQKRLLKELGRPKRPMNAYLRFCAENKP-VVGLSKEDNKMQMKNLGMQWRRLPDAERDRYTKEAEADMKRYQEEMKKWEDKMLASENV
AFUN003837   QKNAKYLSQLTDAQKEEIKLERQQRNEVKIKRDQKRLLKELGRPKRPMNGYLRFCIQNKP-AVGLSKEENKLQMKNLGVQWRGLTDAEKEPFNKQAEADMKRYQEEMKKWEDRMLSSENV
ADIR004850   QKNAKYLSQLTDAQKQELKAERQLRADEKVKRDQKRLLKELGRPKRPLNSFLLFCAQHKP-VSGLNKEENKVQMKNLGLQWKRLPESEKERYTKEAEAEMKRYQEEMKRWEDKMLASENM
FBgn0038805  EERTKYDATLTEEQRAEIKQLKQDLVDAKERRQLRKRVKELGRPKKPASAFLRFIASERINTPQGDKQTYREWHQKTTAKWTRLSDSEKEVYMQESRKEMELYRKAISVWEEKMIRLGHI
consensus    qknaKYlsqLTdaQk-eik-erqqr-e-K-kReqkrmlKeLGRPKrPlngfLrfcaq-kp---glskeenklqmknlgmqWrrlse-ekery-keaeaemkrYqeemk-WEdkMla-enl

AAEL014794   DVIRRKNLILPPTKMDGKTKAPK----------------------------------
AAEL013643   DVIRRKNLILPPTKMDGKTKAPK----------------------------------
ADAC006750   DLVRRKNALLSPAAAAPGGKTRLGKKEQTGAASGRP---AAGGARA-----------
AALB000722   DLVRRKNALLSPAPTPSGGKARTTKKEQPGAASGRP---AAGGARA-----------
AARA011078   IAVRKKNVLLPPSPASVKAKKGGIPVVD-SAPVAKPKK-PTSAARP-----------
AGAP008499   IAVRKKNVLLPPSPASVKAKKGGIPVVD-SAPVAKPKK-PTSAARP-----------
AQUA003532   IAVRKKNVLLPPSPASVKAKKGGIPVVD-SAPVAKPKK-PTSAARP-----------
ASTE007783   EAVRKKNVLLPPSPASTMAKKGGMPVVG-SG--VFTLA-IAGMLLPDWEQFFDEIKW
AFUN003837   EAIRKKNVLLPPSPASSMAKKGGIPVIE-KQ--KKPTS-TT-TARP-----------
ADIR004850   EVVRKRNVLLTPSPASSRARKGPQQVPGNVAAAAKPNKPATSTTRP-----------
FBgn0038805  DVVRHGNLIDPPEPKPRKTLASKDI--------------------------------
consensus    dvvRkkNvllpPspas-kakkg---v----a--grp-------arp-----------
```

Figure S10

A.

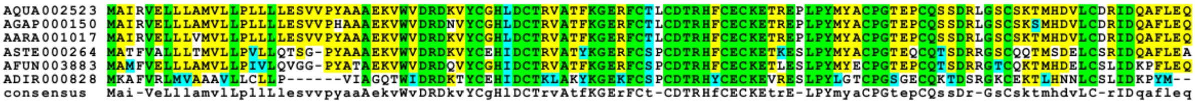


B.

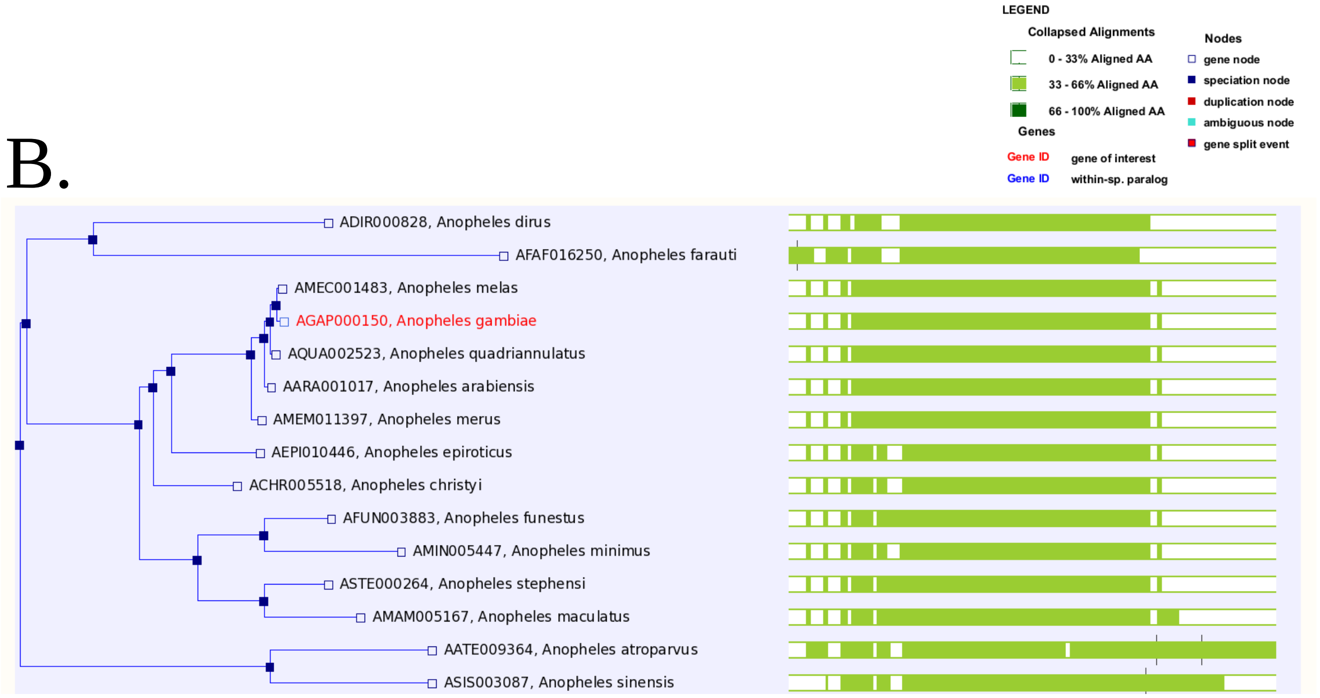

Supplement: Additional file 1: — Figure S1. Adult Anopheles stephensi salivary gland lobe and cellular morphometry. (A) Graph of average length (blue) and width (red) of each lobe from An. stephensi. Ten samples were measured per lobe. Results indicate that lobe length varies more than lobe width, but variations are similar across lobes (standard deviations are shown on the graph). (B) Graph of adult An. stephensi SG cellular morphometry. Each data point represents 50 cellular measurements among 10 lobes. Results show low variability in both female and male gland cellular dimensions, except for proximal lateral cell length, which varies with asymmetric positioning of the duct within that part of the gland. (C) Cross-sections from Nile Red and DAPI stained SGs illustrating the number of nuclei in circumference in the female distal lateral (DL), proximal lateral (PL), and medial lobe (M), and male gland. Proximal (P) and distal (D) are labeled on each lobe/gland for orientation. Arrow in (PL) marks the PL cross-section. The number of circumferential nuclei varies along the length of each lobe. These cell shape and duct observations agree well with previously published electron microscopy cross-sectional views [11]. Scale bars are 50 microns long. Figure S2. Additional details regarding adult Drosophila salivary gland cellular architecture. Adult D. melanogaster (Canton S) SGs stained with DAPI (nuclei), phalloidin (actin), and WGA (chitin; A) or Nile Red (lipids; B-D). A) Evidence of cell turnover was observed, including DNA fragmentation (white arrow), basal cell detachment, and actin accumulation (yellow arrow). (B-D) Female (B) and Male (C-D) SG cell shape. Results show Nile Red staining throughout the cell membranes, highlighting the cuboidal shape of the secretory cells (e.g. Bii, white arrows). Actin is well organized into apico-basal fibers (Biii), and debris can sometimes be visualized in the lumen (Biv, white arrow). The lumenal surface appears irregular [compare Cii to D (proximal portion f [file 13071_2015_1229_MOESM1_ESM.pdf]
